# A Novel Multimodal LC–MS/MS Panel for the Comprehensive Diagnosis of Neurometabolic Disorders in CSF

**DOI:** 10.1002/jimd.70167

**Published:** 2026-04-06

**Authors:** Stine Christ, Julia Rossmann, Sylvia Richter, Sven Garbade, Glynis Klinke, Nenad Blau, Georg Friedrich Hoffmann, Jürgen Günther Okun, Thomas Opladen

**Affiliations:** ^1^ Medical Faculty Heidelberg, Center for Pediatric and Adolescent Medicine Department I Division of Pediatric Neurology and Metabolic Medicine Heidelberg University Heidelberg Germany; ^2^ Metabolomics Core Technology Platform, Centre for Organismal Studies (COS) Heidelberg University Heidelberg Germany; ^3^ Cellzome GmbH A GSK Company Heidelberg Germany; ^4^ Division of Metabolism University Children’s Hospital Zurich Switzerland; ^5^ MetabERN Subnetwork for Neurotransmitters and Other Small Molecules Heidelberg Germany

**Keywords:** CSF, LC–MS/MS, NeuroMetabolom, neurotransmitter, panel, targeted metabolomics

## Abstract

Metabolic testing of cerebrospinal fluid (CSF) is essential for early diagnosis of neurometabolic disorders. However, the large number of differential diagnoses, the phenotypic variance within a clinical picture, and the disease rarity complicate targeted metabolic diagnostics. To improve diagnosis the aim is to establish a liquid chromatography–tandem mass spectrometry (LC–MS/MS) based CSF panel (NeuroMetabolom) which enables a quick, reliable, extensive and cheap method requiring small amounts of analysis material replacing classical single platform analyses. The novel LC–MS/MS NeuroMetabolom method enables measuring all known standard metabolites in CSF, including purines/pyrimidines, sepiapterin, γ‐Aminobutyric acid (GABA), and vitamin B_6_ metabolites, with significantly reduced sample volume and measurement time. Due to their different biochemical interactions several runs with different sample preparation, running time and columns are necessary within the LC–MS/MS. Age‐dependent reference values have been established. Positive controls with consistently detectable metabolite patterns were used to assess analytical reliability. The method has been implemented into routine diagnostics practice. The LC–MS/MS panel enables a simple, comprehensive, and rapid diagnostic approach, reducing time to diagnosis and facilitating early initiation of treatment. Instead of 650 μL, only 250 μL CSF is now required and the analysis time for all metabolites is reduced from 280 to 65.3 min excluding preparation and setup time. Furthermore, the panel is adaptable and can be regularly expanded to include additional clinically relevant metabolites. The novel LC–MS/MS panel, together with a neurometabolomic approach, offers a promising avenue to timely clinical diagnosis.

**Trial Registration:** German Clinical Trials Register: DRKS00007878

**Abbreviations**
2‐OPP: 2S,6S‐2S,6R‐oxopropylpiperidine‐2‐carboxylic acid
3‐MT: 3‐methoxytyramine
3‐OMD: 3‐O‐methyldopa
3‐PHGDH: phosphoglycerate dehydrogenase
5‐HIAA: 5‐hydroxy indoleacetic acid
5‐HTP: 5‐hydroxytryptophan
5‐MTHF: 5‐methyltetrahydrofolate
6 Oxo‐Pip: 6‐oxopiperidine 2‐carboxylic acid
AADC: aromatic L‐amino acid decarboxylase deficiency
ADK: adenosinkinase deficiency
ATQ: antiquitin deficiency
BH4: tetrahydrobiopterin
CSF: cerebrospinal fluid
CT: creatine
DHPR: dihydropteridinereductase
DNAJC12: Syn: non‐phenylketonuric non‐BH_4_‐deficiency hyperphenylalaninemia
ECD: electrochemical detection
FLD: fluorescence detection
GAA: guanidinoacetic acid
GABA: γ‐aminobutyric acid
GAMTD: guanidinoacetate methyltransferase deficiency
GCMS: gas chromatography mass spectrometry
GNMT: glycine‐N‐methyltransferase deficiency
GTPCH: GTP‐cyclohydrolase I deficiency
HPLC/UPLC: high/ultra‐performance liquid chromatography
HVA: homovanillic acid
IEC: ion exchange chromatography
MAT I/III: methionin‐adenosyltransferase I/III deficiency
MS/MS: tandem mass spectrometry
NKH: nonketotic hyperglycinemia
PL: pyridoxal
PLP: pyridoxal‐5‐phosphate
PM: pyridoxamine
PN: pyridoxine
PTPS: 6‐pyruvoyl‐tetrahydropterin synthase deficiency
QC: quality control
RP: reversed‐phase
SAH: S‐adenosylhomocysteine
SAHH: S‐adenosylhomocysteine hydrolase deficiency
SAM: S‐adenosylmethionine
SRD: sepiapterin reductase deficiency
TCA: trichloroacetic acid
THD: tyrosin hydroxylase deficiency
UHPLC: ultra‐high‐performance liquid chromatography
UPLC: ultra‐performance liquid chromatography
UV–VIS: ultraviolet–visible spectroscopy
VLA: vanillic lactic acid

## 1 Introduction

The evaluation of a neurometabolic patient requires a targeted and comprehensive diagnostic work‐up, guided by the clinical presentation and acuity of symptoms. This may include various metabolic tests in urine, plasma and CSF, as well as genetic testing. In recent years, genetic diagnostics have made a significant contribution to improving diagnostics accuracy and have become an integral part of everyday clinical practice [1, 2]. However, the limitations of molecular genetic diagnostics include ambiguous results and variants of unclear significance (VUS), discrepancies between the genotype and clinical phenotypes, and partly, depending on the local facilities, less availability and longer processing time. Furthermore, unexpected results unrelated to the original diagnostic question can pose ethical and counseling challenges [3]. Concomitant metabolic tests should therefore be regarded as complementary and not contradictory to genetic diagnostics in clinical practice, due to their short processing time and reliable results.

In CSF, “basic diagnostics” for amino acids, biogenic amines, 5‐methyltetrahydrofolate (5‐MTHF) and pterins is usually requested to rule out neurometabolic disorders such as nonketotic hyperglycinemia (NKH) or serine synthesis disorders, cerebral folate deficiency, tetrahydrobiopterin (BH_4_) defects, or a primary defect in the synthesis, degradation, or transport of biogenic amines. However, the diagnostic possibilities in CSF are not limited to these parameters. In particular, in children with developmental delays or children with cerebral seizures, additional measurement of other metabolites in CSF—that could also be determined in urine or blood—may be useful to complete the diagnosis and ensure that a rare neurometabolic diagnosis is not overlooked. This includes measuring guanidino compounds (to rule out guanidinoacetate methyltransferase (GAMT) deficiency) [4], pipecolic acid, vitamin B_6_ metabolites [5] (to rule out vitamin B_6_‐dependent epilepsy) and purines/pyrimidines. The determination of S‐adenosylmethionine (SAM) and S‐adenosylhomocysteine (SAH) enables the diagnosis of methionine adenosyltransferase (MAT) I/III deficiency, glycine‐N‐methyltransferase deficiency (GNMT deficiency), S‐adenosylhomocysteine hydrolase deficiency (SAHH deficiency) and adenosine kinase deficiency (ADK) [6, 7].

To date, in most parts of Europe, the selection of metabolic parameters to be analyzed in body fluids remains the responsibility of the requesting physician. However, the presence of multiple differential diagnoses, phenotypic variance within disease patterns, and the rarity of those conditions often complicate targeted metabolic diagnostics [8, 9]. In addition, the requesting physician may have limited experience with metabolic diagnostics. At the same time, early diagnosis and therefore early specific treatment are crucial for the clinical prognosis [10]. Improved diagnostic tools for analyzing metabolites in CSF are therefore needed. Ideally these could include a neurometabolic panel test that enables the analysis of all measurable metabolic parameters in CSF using a unique platform method, replacing the previously used platforms such as ion‐exchange chromatography (IEC), high/ultra‐performance liquid chromatography (HPLC/UPLC), and various detection methods, including fluorescence detection (FLD), electrochemical detection (ECD), ultraviolet–visible spectroscopy (UV–VIS) detection and mass spectrometry. This will reduce the amount of required biomaterial, accelerate turnaround time, minimize preanalytical alterations by limiting freeze–thaw cycles, and replace the stepwise diagnostic approach used to date. Even very rare diseases that the treating physician may not consider can be detected, helping to close existing diagnostic gaps.

The aim of this work was to develop and evaluate an LC–MS/MS‐based “NeuroMetabolom” method that enables reliable analysis of all clinically relevant metabolites in CSF.

Earlier work from our group [11] laid the foundation for this and established a NeuroMetabolom panel. The challenge already at that time was the integration of all metabolites into a single run, which was not possible due to the different structural and physiochemical properties of metabolites (molecular weight, polarity, lipophilicity, aqueous solubility, different charges, size, etc.). Therefore, two runs were established, one for the analysis of a high number of the highly homogenous compounds of purines and pyrimidines by reversed phase (RP)‐UPLC–MS/MS and another run for a selection of chemically different compounds like pyridoxamine (PM), SAM, pyridoxal (PL), pyridoxine (PN), SAH, 3‐methoxytyramine (3‐MT), sepiapterin, 5‐MTHF, vanillic lactic acid (VLA), and tryptamine [11].

Based on these findings, the NeuroMetabolom panel was optimized and extended with the aim to enable a reliable measurement of all clinically relevant CSF metabolites in a single measurement method.

## 2 Methods

### 2.1 Selection of Target Metabolites in CSF

At the beginning of the project, metabolites that define the biochemical fingerprint of individual metabolic disorders and are therefore of high diagnostic value were identified on the various existing analytical platforms (Table 1). These metabolites were designated as target metabolites for a new LC–MS/MS‐based targeted metabolomics approach. In the further course, clinically relevant metabolites were additionally added to the new method.

**TABLE 1:** Overview “old” methods (single platforms) in comparison to the LC–MS/MS panel. The metabolites are combined according to the runs in the LC–MS/MS panel.

	“Old” method—single platforms	NeuroMetabolom LC–MS/MS	Related diseases
Biogenic amines	3‐ortho‐methyldopa^b^, 5‐hydroxyindoleacetic acid^b^, homovanillic acid^b^, 5‐hydroxytryptophan^b^, 3,4‐dihydroxyphenylacetic acid^b^, L‐dopa^b^	3‐ortho‐methyldopa, 5‐hydroxyindoleacetic acid, homovanillic acid, 5‐hydroxytryptophan, 3,4‐dihydrxyphenylacetic acid, l‐dopa, vanillic lactic acid, tryptamine, 3‐methoxytyramine, sepiapterin	Disorders of biogenic amines and pterins (e.g., AADC deficiency, GTPCH or PTPS deficiency, tyrosine hydroxylase deficiency)
Folate metabolism	5‐methyltetrahydrofolic acid^a^	5‐methyltetrahydrofolic acid	Disorders in cerebral folate deficiency
S‐adenosylmethionine, S‐adenosylhomocysteine	Not available	S‐adenosylmethionine, S‐adenosylhomocysteine	Disorders of methylation
Pterins	Neopterin^a^, biopterin^a^	Neopterin, biopterin	Disorders in tetrahydrobiopterin metabolism
Amino acids	Phosphoserine^c^, taurine^c^, phosphoethanolamine^c^, aspartic acid^c^, threonine^c^, serine^c^ Asparagine^c^, glutamic acid^c^, glutamine^c^ a‐Aminoadipic acid^c^, glycine^c^, alanine^c^ Citrulline^c^, a‐aminobutyric acid^c^, valine^c^ Cystine^c^, methionine^c^, isoleucine^c^, leucine^c^ Tyrosine^c^, phenylalanine^c^, γ‐Aminobutyric acid^c^, beta‐alanine^c^, beta‐aminoisobutyric^c^ acid, histidine^c^, 3‐methyl‐histidine^c^, 1‐methyl‐histidine^c^, carnosine^c^, tryptophan^c^, ornithine^c^ Lysine^c^, arginine^c^, hydroxy‐proline^c^, proline^c^ Pipecolic acid^d^	1‐methylhistidine, α‐aminobutyric acid, β‐aminoisobutyric acid, 3‐methylhistidine, 4‐hydroxyproline, acetyltyrosine/N‐acetyltyrosine, adenosylhomocysteine/S‐adenosylhomocysteine, alanine, allo‐isoleucine, α‐aminoadipic acid, anserine, arginine, argininosuccinic acid/argininosuccinate, asparagine, aspartic acid, ß‐alanine, carnosine, citrulline, cystathionine, cysteine sulfate/sulphocysteine, cystine, ethanolamine, γ‐aminobutyric acid, glutamine, glutamic acid, glycine, histidine, homocitrulline, homocystine, hydroxylysine, isoleucine, leucine, lysine, methionine, ornithine, phenylalanine, phosphoethanolamine, phosphoserine, pipecolic acid, proline, saccharopine, sarcosine, serine, taurine, threonine, tryptophan, tyrosine, valine	Disorders of amino acids biosynthesis and breakdown (e.g., phenylketonuria, non‐ketotic hyperglycemia, mitochondriopathy)
B_6_ metabolites	Not available	Pyridoxal‐5‐phosphate, pyridoxal, pyridoxamine, pyridoxine, pyridoxic acid	Vitamin B_6_ dependent epilepsy
Purines and pyrimidines	Not available	Adenine, 2,8‐dihydroxyadenine xanthine, hypoxanthine, inosine, 2‐deoxyinosine, guanosine, 2‐deoxyguanosine, adenosine, 2‐deoxyadenosine, orotic acid, orotidine, uracil, thymine, dihydrouracil, dihydrothymine, ß‐ureidopropionic acid, ß‐ureidoisobutyric acid, pseudouridine, 2‐deoxyuridine, thymidine, 5‐hydroxymethyluracil, allopurinol, AICA riboside, SAICA riboside, SAICAR, succinyladenosine	Disorders of purine/pyrimidine metabolism
Guanidino metabolism	Guanidinoacetate^e^, creatine^e^	Guanidinoacetate, creatine	Disorders in guanidino metabolism

^a^ LC‐FLD: liquid chromatography with fluorescence detection.

^b^ LC‐ECD: liquid chromatography with electrochemical detection.

^c^ IEC‐FLD: ion exchange chromatography with fluorescence detection.

^d^ GC–MS: gas chromatography with mass spectrometry.

^e^ LC–MS/MS: liquid chromatography coupled with tandem mass spectrometry.

### 2.2 Acquisition of Patient Samples

CSF samples from the MetabREF project [approved by the Ethics Committee of Heidelberg University S‐554/2018] were used to establish reference values. In the MetabREF study, children and adolescents having no underlying neurometabolic disease were included for whom CSF, urine and blood samples were obtained for different diagnostic and clinical reasons. Samples were collected in different aliquots with around 0.5–1 mL each and were immediately frozen.

If CSF status was unremarkable (protein, cell count, glucose within normal range, lactate not elevated), the diagnostic residual samples from these patients were used to establish the normal values in this project.

In addition, residual diagnostic samples from the Dietmar Hopp Metabolic Centre were used, in which the extended metabolic diagnostics or the clinical findings also did not reveal any indication of a neurological and/or metabolic underlying disease.

For method verification, pathological residual samples were obtained from patients with a confirmed neurological or metabolic disease in the MetabRND project [approved by the Ethics Committee of Heidelberg University S‐554/2018]. The aim of the MetabRND study was to identify reliable biomarker profiles (biochemical fingerprints) for rare neurological diseases. For this purpose, blood, urine, and CSF were collected together with clinical information from children with various neurological symptoms during routine examinations and stored in a biobank.

In addition, anonymized pathological residual samples from patients with a confirmed diagnosis from the metabolic laboratory in Heidelberg were used.

All studies were conducted in accordance with the current version of the Declaration of Helsinki.

### 2.3 Development of the Analyses

The identified panel of relevant neurometabolites was analyzed in clinical routine prior to the start of the study. Until then, various analytical methods and platforms were required, as this was the only way to analyze such a large number of physiochemically different biomarkers. In total, the selected analytes were analyzed by eight analytical methods using LC–MS/MS, gas chromatography (GCMS), high performance liquid chromatography with fluorescence detection (HPLC‐FLD), high performance liquid chromatography with electrochemical detection (HPLC‐ECD), and IEC. For transferring the selected metabolites to the new LC–MS/MS method, a detailed adaptation according to the chemical and biochemical properties of the various analyses was necessary. Therefore, within the LC–MS/MS method, different analytical “runs” were established (Table 2). The diagnostic sequence is listed in Table 3 with the corresponding metabolites. A detailed method evaluation was carried out for all newly established LC–MS/MS‐based analyses. The details of the method development for the different metabolite groups are given below.

**TABLE 2:** Overview of the final LC–MS/MS panel.

Run	Methods/metabolites	Final 2024	TQS/TQXS	CSF volume	Column	Runtime	Kalibration standards	QC samples
First run	Biogenic amines	RP‐LC‐MS/MS “neurotransmitter”	TQS	25 μL	ACE Excel C18 PFP (100 × 3 mm, 1.7 μm)	12.5 min	7‐point calibration	QC1, 2, 3
	5‐MTHF							
	Sepiapterin, SAM, SAH, VLA, (tryptamin, 3‐MT)							
	SAM and SAH in plasma							
Second run	Pterins	RP‐ LC‐MS/MS method	TQS	100 μL	ACE Excel C18 PFP (100 × 3 mm, 1.7 μm)	8 min	7‐point calibration	QC1, 2, 3
Third run	Amino acids	HILIC‐LC‐MS/MS CS AA Kit	TQXS	25 μL	Chromsystems column	20 min	3‐point calibration	QC1, 2, 3
Fourth run	Vitamin B_6_ metabolites	RP‐LC‐MS/MS, Rossmann et al. 2021	TQS	25 μL	Waters BEH C18 (100 × 2.1 mm, 1.7 μm)	4.8 min	7‐point calibration	QC1, 2, 3
Fifth run	Purines and pyrimidines	RP‐LC‐MS/MS, Klinke et al. [11] (purines and pyrimidines)	TQS	25 μL	ACE Excel C18 PFP (100 × 3 mm, 1.7 μm)	15 min	10‐point calibration	QC1, 2, 3
Sixth run	Guanidino compounds	RP‐LC‐MS/MS, Carling et al. [12] and Haas et al. [4] modified	TQS	50 μL	ACE Excel C18‐AR (100 × 3 mm, 1.7 μm)	5 min	8‐point calibration	QC1, 2

**TABLE 3:** Diagnostic sequence of NeuroMetabolom and its development.

Run	Methods/metabolites	Platform (start 2019)	Developments 2020–2022	Developments 2023–2024	Final 2024
First run	Biogenic amines	RP‐HPLC‐ECD (Hyland [13], 1986; Bräutigam 2001)	RP‐LC‐MS/MS metabolites were added in fifth run, measured as described in Klinke et al. [11] (purines and pyrimidines)	RP‐LC‐MS/MS Klinke et al. [11] modified mobile phases changed in 0.2% formic acid and acetonitrile column ACE Excel C18PFP, 25°C samples were diluted 1:4, stabilization buffer instead of water	RP‐LC‐MS/MS Klinke et al. [11] modified
	5‐MTHF	RP‐HPLC‐FLD (Ormazabal 2006) RP‐LC‐MS/MS (Klinke et al. [11]) (H‐mix)			
	Sepiapterin, SAM, SAH, VLA, (tryptamin, 3‐MT)	RP‐LC‐MS/MS (Klinke et al. [11]) (H‐mix)			
	SAM and SAH in plasma	Not measured			
Second run	Pterines	RP‐HPLC‐FLD Blau, Hyland		RP‐LC‐MS/MS Klinke et al. [11] modified samples were oxidized with MnO_2_ like before, diluted 1:3 with IS‐mix	RP‐LC‐MS/MS Klinke et al. [11] modified
Third run	Amino acids	IEC AA Kit Fa. Onken		HILIC‐LC‐MS/MS CS AA Kit	HILIC‐LC‐MS/MS CS AA Kit
Fourth run	Vitamin B_6_ metabolites	RP‐LC‐MS/MS Klinke et al. [11] (H‐mix)	RP‐LC‐MS/MS Rossmann et al. 2021		RP‐LC‐MS/MS Rossmann et al. 2021
Fifth run	Purines–pyrimidines	RP‐LC‐MS/MS Klinke et al. [11] (purines and pyrimidines)	Metabolites of first run were included, no analytical changes	RP‐LC‐MS/MS Klinke et al. [11] (purines and pyrimidines)	RP‐LC‐MS/MS Klinke et al. [11] (purines and pyrimidines)
Sixth run	Guanidino compounds	RP‐LC‐MS/MS Carling et al. [12] and Haas et al. [4] modified			RP‐LC‐MS/MS Carling et al. [12] and Haas et al. [4] modified

### 2.4 Analytical Methods

#### 2.4.1 First Run: Biogenic Amines, Biogenic Amines Metabolites and Sepiapterin in CSF, SAM, and SAH in CSF and Plasma

The measurement of SAM, SAH, sepiapterin, 5‐MTHF, VLA, GABA, homo vanillic acid (HVA), 5‐hydroxy‐indol‐acetic acid (5‐HIAA), 3‐ortho‐methyldopa (3‐OMD), 5‐hydroxy tryptophan (5‐HTP), and L‐Dopa was developed using an RP‐LC–MS/MS method using a Waters Acquity UPLC system connected with a Waters Xevo TQ‐S tandem mass spectrometer (MS/MS).

Standard substances of analytes and internal standard substances were purchased from CDN Isotopes, Sigma, Merck, Roth, Cayman Chemicals, and TCI Europe.

Stock solutions of 3‐MT (1 mM), sepiapterin (1 mM), VLA (1 mM), GABA (10 mM) were prepared in water, of 3‐OMD (5 mM), 5‐HIAA (2,5 mM), 5‐HTP (5 mM), 5‐MTHF (5 mM), L‐Dopa (5 mM), HVA (5 mM) in 5 mM ascorbic acid and of SAH (1 mM) and SAM (1 mM) in 1 M HCl.

Internal standards were isotopically labeled in the same way as the analytes. Stock solutions of 3‐OMD‐D_3_ (1 mM), 5‐HIAA‐D_6_ (1 mM), 5‐HTP‐D_3_ (1 mM) and HVA‐D_5_ (1 mM) were prepared in MeOH, of ^13^C_5_‐5‐MTHF (2,5 mM), L‐Dopa‐D_3_ (1 mM), PN‐D_2_ (1 mM) in water, of SAH‐D_4_ (1 mM) and SAM‐D_3_ (1 mM) in 1 M HCl. IS stock solutions were combined in the IS‐solution containing 50 nM of 3‐OMD‐IS, 5‐HIAA‐IS, 5‐MTHF‐IS, L‐Dopa‐IS, PN‐IS, SAH‐IS, and SAM‐IS, 100 nM of 5‐HTP‐IS, and 500 nM of HVA‐IS.

For MS/MS conditions, see Table S1. Chromatography was realised using an ACE Excel C18‐PFP (100 × 3 mm, 1.7 μm, EXL‐1710‐1003U, MZ‐Analysentechnik GmbH) kept at 25°C. Mobile phases consisted of mobile phase A with aqueous 0.2% formic acid and mobile phase B was acetonitrile. For sample preparation 25 μL of CSF was diluted 1:4 by adding 25 μL IS‐solution and 50 μL stabilization buffer (Stabilization Buffer, Chromsystems, order no: 80875). The runtime was 12.5 min and injection volume 10 μL. The samples were quantified by a seven‐point calibration with two different concentration ranges (Table S2).

For quality control, three concentration levels, low, mean, and high range were selected for all analytes (Table S2). The reference values were established by analyzing the data of at least 579 CSF samples.

#### 2.4.2 Second Run: Pterins in CSF

A new measurement method for the pterins neopterin and biopterin was realized using mass spectrometry detection. For sample preparation 100 μL CSF and 0.5 mg MnO_2_ were vortex mixed with 10 μL aqueous trichloroacetic acid (TCA) 30% and incubated for 5 min and finally centrifuged. Fifty microliteres of the supernatant was mixed with 100 μL of diluted IS‐mix containing 50 nM Neo‐IS and Bio‐IS. A 7‐point calibration was prepared with pooled CSF that was spiked with 1.56, 3.13, 6.25, 12.5, 25, 50, and 100 nM for biopterin and 3.12, 6.26, 25, 50, 100, and 200 nM for neopterin, aliquoted and stored at −80°C. The measurement was performed with a Waters Acquity chromatography system coupled to a Waters Xevo TQ‐S MS/MS. For chromatographic separation an ACE Excel C18‐PFP column (100 × 3.0 mm; 1.7 μm, EXL‐1710‐1003U, MZ‐Analysentechnik GmbH), mobile phase A consisting of 0.2% aqueous formic acid and mobile phase B consisting of acetonitrile was used. The run time was 8 min, the flow rate was 300 μL/min and the injection volume 10 μL. The gradient with mobile phase A was as follows: 0–0.8 min, 99%; 5.0 min, 80%; 5.5–6.0 min, 1%; 7.0–8.0 min, 99%.

The analytes were detected by multiple reaction monitoring (MRM) in positive ionization mode, capillary voltage was 0.7 kV, desolvation temperature 600°C at a gas flow of 900 L/h and cone gas flow 180 L/h (MS/MS conditions for single reaction monitoring, see Table S1). Argon was used for collision gas at a flow rate of 0.14 mL/min. Detection took place between 1.5 and 5.0 min, but flow was otherwise set to waste.

Neopterin, biopterin, 1 M NaOH, 1 M HCl and TCA were purchased from Sigma‐Aldrich Chemie GmbH. ^13^C_5_neopterin (Neo‐IS) and biopterin‐ d3 (Bio‐IS) were purchased from LGC Standards. Acetonitrile and formic acid were purchased from Biosolve (Valkenswaard, the Netherlands).

Stock solutions (1 mM) of biopterin and Bio‐IS prepared in ultrapure water (0.2 μm barnstead filter, GENPURE PRO filter (Thermo Scientific, Waltham, MA)). Neopterin and Neo‐IS were dissolved in NaOH (1 mM). From those stock solutions, a mix of neopterin (10 μM) and biopterin (5 μM) was prepared for calibration and controls.

An internal deuterated standard mix (IS) of 5 μM Neo‐IS and Bio‐IS was prepared in HCl (10 mM) and stored at −80°C. Before each run, the IS‐mix was diluted by a factor of 100 with milliQ water and was used for sample preparation.

Three levels of quality control (QC) were prepared, QC low was pooled CSF, QC mean was 14 mL pooled CSF that was spiked with 75 μL of the mix neopterin/biopterin and QC high was 14 mL pooled CSF that was spiked with 150 μL of the mix neopterin/biopterin with 10 μM neopterin and 5 μM biopterin (for intra‐ and interday data, see Supporting Information). All QC's were aliquoted and stored at −80°C.

#### 2.4.3 Third Run: Amino Acids in CSF (Using Chromsytems Kit)

The measurement of 48 amino acids in CSF samples was performed by a commercially available kit for amino acid analysis in plasma samples of the Chromsystems company (Gräfelfing, Germany) that was modified for the analysis of amino acids in CSF. Thereby, all standards and quality control samples were diluted with water by a factor of 10, while the sample preparation procedure, buffers, reagents, and mobile phases A and B of the commercial kit remained as described in the kit instructions. For measurement, a Waters Acquity UPLC system was coupled to a Waters Xevo TQ‐XS MS/MS. Chromatographic separation was performed using an analytical column from the Chromsystem amino acid analysis kit at room temperature and a flow rate of 0.3 mL/min to 1.8 mL/min for mobile phases A and B during a run time of 20.5 min. A maximum pressure of 280 bar was recommended. For sample preparation, 25 μL of CSF were pipetted into a deep‐well plate and added by 50 μL reconstituted internal standard mixture and 400 μL precipitation reagent, heat sealed with a peelable aluminum foil, and mixed with a thermo shaker for 2 min at 1200 U/min. The deep‐well plate is then centrifuged for 5 min at 2000 × *g*_max_ and the peelable foil is removed carefully. Two‐hundred microliters of supernatant is pipetted into a collection plate and heat sealed with a pierceable and peelable aluminum foil for measurement. Injection volume is optimized to 4 μL. Quantification was done with a 3‐point calibration curve that was diluted 10‐fold with water with individual concentration of all amino acids. Quality control was realized with three levels in low, mean, and high concentration that were also diluted 10‐fold with water. All amino acids, except glycine, were quantified using a linear calibration regression with weighting of 1/x. Glycine was quantified using a slightly different calibration, whereby the calibration was set to zero using the ‘force 0’ function. This makes it possible to detect reductions as well. Further information of amino acid concentrations of 10‐fold diluted calibration standards and 10‐fold diluted quality control samples are available in Tables S3–S6. Linear regression was above 0.99 or higher.

#### 2.4.4 Fourth Run: Vitamin B_6_ Metabolites

The measurement of the vitamin B_6_ metabolites PM, PN, PL, pyridoxal‐5‐phosphate (PLP) and pyridoxic acid (PA) were performed by UHPLC–MS/MS according to Rossmann et al. [14], except for a smaller amount of required CSF due to improved autosampler settings.

#### 2.4.5 Fifth Run: Purines and Pyrimidines in CSF

The measurement of pyrimidines, purines, and others was performed with RP‐LC–MS/MS using a Waters Acquity UPLC system connected with a Waters Xevo TQ‐S MS/MS and was previously published [11]. As mentioned above, also in this method we could reduce the amount of required CSF to 25 μL due to improved autosampler settings; the dilution ratio with 1:4 remained the same.

#### 2.4.6 Sixth Run: Guanidino Compounds in CSF

Measurement of guanidinoacetic acid (GAA) and creatine (CT) were performed with RP‐LC–MS/MS using a Waters Acquity UPLC system connected with a Waters Xevo TQ‐S MS/MS and modified according to Haas et al. and Carling et al. [4, 12]. For MS/MS conditions, see Table S1. Chromatography was realized using an ACE Excel C18‐AR (100 × 3 mm, 1.7 μm, EXL‐179‐1003U, MZ‐Analysentechnik GmbH) column with prefilter and an isocratic gradient with 0.2 mL/min with a column temperature of 40°C. Mobile phase A consisted of 0.4% aqueous formic acid. For sample preparation 50 μL of CSF was diluted with 100 μL of aqueous IS‐solution containing 1 μM GAA‐IS and 30 μM CT‐IS and vortex mixed for 10 s, incubated for 10 min and subsequent centrifuged. Fifty microliters of extract was diluted with 150 μL of ultrapure water. The runtime was 5 min and injection volume 1 μL. The samples were quantified by an eight‐point calibration from 0.075 to 4.8 μmol/L GAA and 3 to 192 μmol/L CT prepared in Ringer solution. For quality control, two concentrations in low and mean range were selected for all analytes respectively. The concentrations ranged from QC1 with 0.75 μM GAA and 30 μM CT to QC2 with 1.5 μM GAA and 60 μM CT. The isotopically labeled analogues of CT and GAA, CT‐D_3_ (D‐3689) and GAA‐D_2_ (D‐6320) were used as internal standards and were both provided by CDN Isotopes.

### 2.5 Statistical Analysis

R language version 4.5.1 was used for statistical analysis. To estimate references ranges for SAM/SAH in CSF, vitamin B6 metabolites, biogenic amines, biogenic amines metabolites, pterins, and guanidino compounds, a three‐step procedure was applied: First, values below the 0.5% and above the 99.95% percentile were treated as extreme outliers and purged from the data. Second, quantile regression with lambda, mu and scale (LMS) was applied: For each metabolite, to best obtain normality of the response, three quantile regression models with Box–Cox to normal, Box–Cox to gamma and Box‐Cox to Yeo‐Johnson transformation was applied. If more than one model successfully converged, the Akaike Information Criteria (AIC) was used to select the quantile regression model with the lowest AIC, and the lower 2.5% and upper 97.5 percentile curves were estimated from this model and suggested as lower and upper reference levels. For some metabolites (e.g., 1‐methylhistidine, proline), no quantile regression model converged and the 97.5% percentile was used an upper reference level. Third, the statistically suggested reference ranges were further evaluated by clinical and laboratory physicians in terms of practical and clinical feasibility and adjusted where necessary, for example, rounded to zero decimal places. The VGAM package version 1.1–14 was used to apply LMS quantile regression [15].

## 3 Results

The study focused on adapting individual analytical methods to enable the measurement of all identified standard metabolites using a common LC–MS/MS platform. It was conducted between March 2017 and December 2024 in a multidisciplinary setting involving physicians, biologists, chemists, and technicians.

With the aim of analyzing all identified target metabolites in a single comprehensive run, the methodology of the recently described LC–MS/MS method of Klinke et al. [11] was modified. However, it soon became apparent that the physical and chemical differences between the various metabolites were too significant to be measured in a single LC–MS/MS run, meaning that different, separate LC–MS/MS‐based runs had to be established to measure the entire metabolite spectrum.

The finally established LC–MS/MS NeuroMetabolom panel consists of six different runs, requires 250 μL CSF in total and the run time excluding preparation and setup time takes 65.3 min for complete analysis of all metabolites. Where available, fraction 3 or 4 was used in most samples. However, in a few random samples, we were unable to see any relevant difference between the individual fractions.

For all methods, a detailed method evaluation was performed, and reference values were established. The statistical evaluation didn't show a gender dependency of the metabolites.

The diagnostic performance of the respective measurement methods was demonstrated using pathological samples that revealed the expected biochemical patterns (Tables 4, 5, 6, 7, 8, 9, 10).

**TABLE 4:** Confirmation of known neurotransmitter disorders: Biogenic amines concentration [nmol/L] in CSF of patients with AADC (aromatic L‐amino acid decarboxylase deficiency), TH (tyrosine hydroxylase deficiency), DNAJC12 (Syn: non‐phenylketonuric non‐BH_*4*_‐deficiency hyperphenylalaninemia), PTPS (6‐pyruvoyl‐tetrahydropterin synthase deficiency) and SRD (sepiapterin reductase deficiency) determined by the new LC–MS/MS (MS/MS) method and by HPLC‐ECD (ECD) method.

Disease	AADC	AADC	TH	TH
Medication	b.t.	u.t.	b.t.	b.t.
Sex	m	f	f	f
Age	4 m	3 y 6 m	1 y 5 m	1 y 6 m

	MS/MS	ECD	MS/MS	ECD	MS/MS	ECD	MS/MS	ECD
HVA	27 ↓	19 ↓	87 ↓	80 ↓	12 ↓	15 ↓	72 ↓	123 ↓
5‐HIAA	4 ↓	95 ↓	3 ↓	7 ↓	241	142 ↓	394	305
3‐OMD	3493 ↑	2761 ↑	1237 ↑	1025 ↑	9	10	8	16
5‐HTP	225 ↑	243 ↑	112 ↑	142 ↑	5	5	10	0
L‐Dopa	281 ↑	147 ↑	127 ↑	111 ↑	0	1	1	3
VLA	243 ↑	n.m.	60 ↑	n.m.	1	n.m.	1	n.m.
GABA	29	n.m.	96	n.m.	36	n.m.	42	n.m.
Sepiapterin	0	n.m.	0	n.m.	0	n.m.	0	n.m.
HVA/5‐HIAA	6.8 ↑	0.2 ↓	29.0 ↑	11.4 ↑	0.1 ↓	0.1 ↓	0.2 ↓	0.4 ↓

Disease	DNAJC12	PTPS	PTPS	SRD
Medication	u.t.	b.t.	b.t.	u.t.
Sex	f	f	m	m
Age	7 y 10 m	20 days	1 y 1 m	16 y 4 m

	MS/MS	ECD	MS/MS	ECD	MS/MS	ECD	MS/MS	ECD
HVA	125 ↓	170 ↓	14 ↓	11 ↓	128 ↓	146 ↓	49 ↓	90 ↓
5‐HIAA	54 ↓	50 ↓	4 ↓	n.d. ↓	53 ↓	39 ↓	25 ↓	26 ↓
3‐OMD	511 ↑	446 ↑	76	64	23	13	393 ↑	408 ↑
5‐HTP	124 ↑	115 ↑	5	0	3	1	23 ↑	137 ↑
L‐Dopa	12	7	2	4	1	3	n.m.	17 ↑
VLA	8	n.m.	4	n.m.	1	n.m.	9	n.m.
GABA	80	n.m.	22	n.m.	40	n.m.	n.m.	n.m.
Sepiapterin	0	n.m.	0	n.m.	0	n.m.	10	n.m.
HVA/5‐HIAA	2.3	3.4	3.5	—	2.4	3.7	2.0	3.5

*Note:* Medication: before treatment (b.t.), under treatment (u.t.). Sex: male (m), female (f). Age: age of sampling, months (m), years (y); n.m., not measured; n.d., not detected.

**TABLE 5:** Confirmation of known folate disorders: 5‐MTHF concentration [nmol/L] in CSF of patients with MTHFR‐deficiency (5‐methyltetrahydrofolate reductase), HFM (hereditary folate malabsorption) and CFD (cerebral folate deficiency) determined by the new LC–MS/MS (MS/MS) method and by HPLC‐FLD (FLD) method.

Disease	MTHFR‐deficiency	HFM	CFD	MTHFR‐deficiency
Medication	b.t.	u.t.	u.t.	u.t.
Sex	m	m	f	f
Age	2 m	1 y 7 m	3 y 7 m	1 y 4 m

	MS/MS	FLD	MS/MS	FLD	MS/MS	FLD	MS/MS	FLD
5‐MTHF	23 ↓	16 ↓	13 ↓	10 ↓	26 ↓	22 ↓	4 ↓	n.d. ↓

*Note:* Medication: before treatment (b.t.), under treatment (u.t.). Sex: male (m), female (f). Age: age of sampling, months (m), years (y).

**TABLE 6:** Confirmation of known pterins disorders: Pterins concentration [nmol/L] in CSF of patients with DHPR (Dihydopteridinereductase), GTPCH (GTP‐cyclohydrolase I deficiency) and PTPS (6‐pyruvoyl‐tetrahydropterin synthase deficiency) determined by the new LC–MS/MS (MS/MS) method and by HPLC‐FLD (FLD) method.

Disease	DHPR	DHPR	GTPCH	GTPCH	PTPS	PTPS
Medication	b.t.	b.t.	u.t.	u.t.	b.t.	b.t.
Sex	m	f	m	m	f	f
Age	1 m	5 y 1 m	3 y 10 m	2 y 10 m	16 d	6 d

	MS/MS	FLD	MS/MS	FLD	MS/MS	FLD	MS/MS	FLD	MS/MS	FLD	MS/MS	FLD
Neopterin	40.5 ↑	39↑	14	15	0↓	0↓	0↓	2↓	203↑	194↑	396↑	354↑
Biopterin	79.3 ↑	97↑	59.4↑	46↑	0↓	1↓	5.2↓	12	9.3	10	13.3	14
% B	66	71	81	75	—	—	—	86↑	4↓	5↓	3↓	4↓

*Note:* Medication: before treatment (b.t.), under treatment (u.t.). Sex: male (m), female (f). Age: age of sampling, days (d), months (m), years (y).

**TABLE 7:** Confirmation of known amino acid disorders: Amino acid concentration [μmol/L] in CSF of patients with DHPR (dihydopteridinereductase), NKH (nonketotic hyperglycinemia), asparagine‐ and glutamine synthetase deficiency, 3‐PHGDH deficiency and ATQ (antiquitin deficiency).

Disease	NKH	NKH	DHPR	DHPR	Asparagine synthetase deficiency	Glutamine synthetase deficiency	3‐PHGDH‐deficiency	ATQ	ATQ
Medication	u.t.	u.t.	u.t.	u.t.	b.t.	b.t.	b.t.	b.t.	b.t.
Sex	f	f	f	f	f	m	m	f	f
Age	4 d	7 m	14 y 18 d	11 y, 11 m, 24 d	153 d	2 y, 10 d	13 y, 7 m, 4 d	4 m	5 d
1‐Methylhistidine	1.9 ↑	0.2	0.5	0	0.5	0.4	0.6	0.4	2.6 ↑
Alpha‐aminobutyric acid	5.5 ↑	3.2	3.1	2.4	1.9	2.5	1.8	3.8	29.1 ↑
Beta‐aminoisobutyric acid	0.2 ↑	0.1	0	0	0.1	0	0	0.1	0
3‐Methylhistidine	0.6	0.	0.29	0.3	0.2	0.5	0.4	0.5	3.8 ↑
4‐Hydroxyproline	2.6 ↑	0.9	0.8	0.6	1.3	0.4	0.5	1.8	4.2 ↑
Acetyltyrosine	0	0	0	0	0	0	0	0.1	0
Adenosylhomocysteine	0.0	0	0	0	0	0.1	0.1	0	0.1
Alanine	26.7	25.2	19.3	22.8	25.3	43.9 ↑	16.9	43.2 ↑	61.4 ↑
Allo‐Isoleucine	0.4	0.3	0.2	0.5	0.2	0.3	0.1	0.2	1.1 ↑
Alpha‐aminoadipic acid	0.1	0.2	0.1	0.1	0.2	0.3 ↑	0.1	0.1	0.1
Anserine	0	0	0	0	0	0	0	0	0
Arginine	20.8	19.2	18.9	24.6	24.1	19.8	18.6	21.2	12.0
Argininosuccinic acid	0.4	0.1	0.1	0.3	0.2	0.7 ↑	0.3	0.2	0.4
Asparagine	17.2 ↑	7.3	5.0	6.7	2.6 ↓	5.0	4.8	9.0	22.3 ↑
Aspartic acid	0.8	0	0.5	0.5	0.5	1.1 ↑	2.0 ↑	0	0
Beta‐alanine	0.1	0.2	0.1	0.1	0.3	0.2	0.1	00	0.7 ↑
Carnosine	0.1	0.	0.1	0.1	0.3 ↑	0.2	0.1	0.2	0.1
Citrulline	10.4 ↑	3.4	1.5	2.7	1.3	1.8	1.9	3.2	5.0 ↑
Cystathionine	0.4	0.5	0	0	0.1	0.2	0	0.3	0.5
Cysteine sulfate	0.1	0.2	0.1	0.1	0.1	0.1	0.1	0.2	0.2
Cystine	0.2	0.1	0	0	0.1	0.3	0.2	0	0.1
Ethanolamine	80.9 ↑	7.1	15.3	13.3	12.7	11.1	11.2	14.9	141.0 ↑
Gamma‐aminobutyric acid	0.1	0	0.2	0.2	0.1	0.4	0.4	0	0.8
Glutamine	1022 ↑	363	521	524	301	227 ↓	360	403	763 ↑
Glutamic acid	0	0.8	0.4	0.8	5.0 ↑	3.1 ↑	5.1 ↑	0.3	10.2 ↑
Glycine	127.5 ↑↑↑	55.7 ↑	5.5	8.2 ↑	7.2	7.0	5.3	7.3	10.2 ↑
Histidine	29.3 ↑	10.9	13.8	18.3	1.2	14.7	10.0	19.0	47.7 ↑
Homocitrulline	0.1	0.1	0	0	0	0	0	0.1	0.3 ↑
Homocystine	0	0	0	0	0	0	0	0	0
Hydroxylysine	0	0	0	0	0	0.3 ↑	0	0	0
Isoleucine	4.4	5.8	3.8	6.9	6.0	4.9	3.9	7.7	14.0 ↑
Leucine	17.3	12.6	12.0	21.5	15.4	11.8	13.0	15.8	25.9 ↑
Lysine	21.7	1.4	19.7	10.9	18.0	19.8	14.1	0	0
Methionine	6.6 ↑	3.3	3.5	3.0	2.9	1.2	1.4	5.2	23.2 ↑
Ornithine	6.8	5.7	4.1	5.5	8.8	8.8	3.6	14.4 ↑	23.6 ↑
Phenylalanine	23.5 ↑	7.6	23.9 ↑	32.5 ↑	9.5	6.0	8.7	11.9	41.8 ↑
Phosphoethanolamine	4.3	1.9	4.5	8.2	2.0	3.5	2.0	3.7	6.1
Phosphoserine	0	0	0	0	0	0.6	0	0	0
Pipecolic acid	0.1	0.1	0	0	0	0	0	2.5 ↑	13.8 ↑↑↑
Proline	4.0 ↑	1.6	0.7	0.5	1.3	0.9	0.8	1.4	6.2 ↑
Saccharopine	0.7 ↑	0.3	0.2	0.2	0.2	0.3 ↑	0.2	0.3	2.8 ↑
Sarcosine	0	0	0	0	0	0	0	0	0
Serine	58.3	37.1	35.8	29.1	37.2	60.7 ↑	10.0 ↓	76.0 ↑	128.8 ↑
Taurine	13.6	9.7	8.4	7.2	6.5	8.9	5.4	10.1	28.3 ↑
Threonine	74.5 ↑	36.8	46.7	27.7	45.0	21.5	30.4	63.0	105.3 ↑
Tryptophan	4.7	2.4	2.7	2.7	2.7	1.6	1.8	4.0	6.6 ↑
Tyrosine	19.2 ↑	9.0	7.7	7.1	10.7	7.5	7.9	19.3	35.5 ↑
Valine	19.2 ↑	18.4	14.3	15.5	12.4	11.7	12.3	15.0	42.4 ↑

*Note:* Medication: before treatment (b.t.), under treatment (u.t.). Sex: male (m), female (f). Age: age of sampling, days (d), months (m), years (y).

**TABLE 8:** Confirmation of known antiquitin deficiency: Results of four patients with a vitamin B_6_ dependent epilepsy; ATQ (antiquitin deficiency).

Disease	ATQ	ATQ	ATQ	ATQ
Medication	b.t.	b.t.	b.t.	u.t.
Sex	m	f	f	m
Age	6d	5d	4 m	9d
PM	0	0.4	0	13.5
PL	< 5	13.9	7.8	2666.7
PN	0	0	0.9	3439.3
PLP	6.7	1.3	8.4	58.7
PA	0	0	0	71.6
PLP/PL	—	0.094	1.07	0.02

*Note:* Medication: before treatment (b.t.), under treatment (u.t.). Sex: male (m), female (f). Age: age of sampling, days (d), months (m), years (y).

**TABLE 9:** Reference values and confirmation of known purines and pyrimidines disorders: Purines and pyrimidines concentrations [μmol/L] in CSF of patients with LNS (Lesch Nyhan syndrome), ADSL (adenosyl‐succinatelyase deficiency) and MoCD (molybdenum cofactor deficiency) determined by LC–MS/MS method.

Disease		LNS	LNS	ADSL	MoCD A
Medication		b.t.	b.t.	b.t.	b.t.
Sex		f	m	m	f
Age		5 m	1 y 5 m	7 d	5 m
2‐Desoxyadenosine	0–0.4	0	0	0	0
2‐Desoxyinosine	0–0.2	0	0	0	0
2‐Desoxyuridine	0–0.4	0.2	0.1	0.2	0.1
5‐Hydroxymethyluracil	0–4.0	0	0	0	0
Adenin	0–2.0	0	0	0	0
Adenosine	0–0.2	0	0	0.2	0
Allopurinol	0–0.2	0	0.1	0	0
ß‐ureidoisobutyric acid	0–2.0	0	0	0	0
ß‐ureidopropionic acid	0–0.3	0.1	0.1	0.1	0
Dihydrothymine	0–1.2	2.4 ↑	1.0	0.9	1.1
Dihydrouracil	0–5.0	4.5	3.3	4.5	1.7
Guanosin	0–0.2	0.1	0.1	0	0
Hypoxanthine	0–4.7	7.3 ↑	33.7 ↑	2.4	6.0 ↑
Inosine	0–1.0	0.8	0.9	0.6	0.9
Orotic acid	0–2.0	0	0	0	0
Orotidin	0–2.0	0.3	1.6	0.2	0.1
Pseudouridin	0–23.1	2.5	2.5	5.9	2.9
Succinyladenosin	0–2.2	1.4	0.7	74.5 ↑	0.4
Thymidin	0–2.0	0	0	0	0
Thymin	0–0.2	0	0	0	0
Uracil	0–0.3	0.3	0.4 ↑	0.2	0.1
Xanthin	0–4.2	4.7 ↑	11.2 ↑	5.2 ↑	10.0 ↑
2‐Desoxyguanosine	0–0.2	0	0	0	0
AICA riboside	0–0.2	0	0.5 ↑	0.1	0
SAICA‐riboside	0–0.4	0.1	0.5 ↑	457.9 ↑	0
SAICAR	0–0.4	0	0	0	0

*Note:* . Medication: before treatment (b.t.), under treatment (u.t.). Sex: Male (m), female (f). Age: age of sampling, days (d), months (m), years (y).

**TABLE 10:** Confirmation of known S‐adenosylmethionine (SAM) and S‐adenosylhomocysteine (SAH) associated disorders: Concentrations of SAM and SAH [nmol/L] and homocysteine and methionine [μmol/L] in plasma of patients with ADK (adenosine kinase deficiency), 5‐MTHF (5‐MTHF deficiency), HCY (homocystinuria); MAT I (methionine adenosyltransferase I/III‐deficiency); SAHH (S‐adenosylhomocysteine hydrolase deficiency).

Disease	ADK	5‐MTHF	NSUN3 deficiency	SAHH	HCY	Hepatopathy
Medication	u.s.t.	b.t.	SAM	n.k.	u.t.	b.t.
Sex	m	m	m	f	m	f
Age	4 y 1 m	2 m	2 y 11 m	2 y 8 m	5 y	1 y 1 m
SAM	122 ↑	42	154 ↑	135 ↑	459 ↑	541
SAH	107 ↑	94 ↑	22	127 ↑	108 ↑	100
SAM/SAH ratio	1.1	0.5	7	1.1	4.25 ↑	5.4
Homocysteine	7	206 ↑	11	3.6	150 ↑	14
Methionine	27	7 ↓	19	n.m.	1252 ↑	274

Disease	MAT I			MAT I	
Medication	n.t.			n.t.	
Sex	f			f	
Age	10 m	2 y 2 m	2y 9 m	5 y 4 m	6y 8 m
SAM	49	134	53	84	59
SAH	41	15.8	15.7	11.5	13
SAM/SAH Ratio	1.2	8.5	3.4	7.3	4.6
Homocysteine	15	14	9	6	9
Methionine	104	70	68	108	132

*Note:* Medication: before treatment (b.t.), under treatment (u.t.), under symptomatic treatment (u.s.t.), no treatment (n.t.), not known (n.k.). Sex: Male (m), female (f). Age: age of sampling, days (d), months (m), years (y).

### 3.1 Detailed Results of Each Measurement Method

The following paragraph describes the individual methods in detail, shows the results of the method evaluation, and provides examples of clinical application. A detailed overview of detectable diseases can be found in Table 1.

Samples have to pass through the following runs:

#### 3.1.1 First Run: Biogenic Amines, Biogenic Amines Metabolites and Sepiapterin in CSF, SAM, and SAH in CSF and Plasma

Age‐appropriate reference values were determined for the metabolites of the first run (HVA, 5‐HIA, 3‐OMD, L‐Dopa, 5‐OH‐tryptophan, VLA, GABA, sepiapterin, and 5‐MTHF) using 579 CSF samples (Table 11). In order to verify the validity and comparability of the new method with the established method, 102 samples from known patients and patients whose diagnosis was unclear were subsequently examined using the newly established LC–MS/MS method. The results of biogenic amines and 5‐MTHF correlated very well with the previous measurements and were always within the target range in external quality assurance schemes. In known patients, the expected pathologic pattern was reliably seen (Table 4).

**TABLE 11:** Reference values for biogenic amines and 5‐MTHF: Reference values [nmol/L] in CSF samples for analytes of the neurotransmitter method determined by the measurement and data analysis of 579 CSF samples.

Age	nmol/L									
	HVA	5‐HIAA	HVA/5‐HIAA	3‐OMD	5‐HTP	L‐Dopa	VLA	GABA	Sepiapterin	5‐MTHF
0–90 d	418–1306	345–970	0.9–2.2	0–195	0–28	0–15	0–13	0–27	0–5	69–223
91–183 d	397–1047	203–694	1.3–2.5	0–120	0–22	0–15	0–10	0–55	0–5	69–202
184–365 d	373–1019	176–655	1.4–2.6	0–60	0–20	0–15	0–10	0–100	0–5	61–192
1–2 y	347–984	146–605	1.5–3.4	0–60	0–20	0–15	0–10	0–100	0–5	51–178
3–5 y	294–860	98–424	1.9–3.7	0–60	0–20	0–15	0–10	0–140	0–5	38–131
6–8 y	252–738	86–316	1.8–3.7	0–60	0–20	0–15	0–10	0–140	0–5	38–131
9–12 y	194–631	77–287	1.7–3.7	0–60	0–20	0–15	0–10	0–140	0–5	35–111
13–15 y	150–469	66–250	1.4–3.2	0–60	0–20	0–15	0–10	0–140	0–5	32–97
16–199 y	93–368	47–215	1.3–2.9	0–60	0–20	0–15	0–10	0–140	0–5	32–97

Abbreviations: 3‐OMD, 3‐O‐methyldopa; 5‐HIAA, 5‐hydroxy indoleacetic acid; 5‐HTP, 5‐hydroxytryptophan; 5‐MTHF, 5‐methyltetrahydrofolate; d, days (age); GABA, γ‐aminobutyric acid; HVA, homovanillic acid; VLA, vanillic lactic acid; y, years.

Sepiapterin was significantly elevated in one case of known sepiapterin reductase deficiency (SRD) (10 nmol/L, range 0–5 nmol/L) while all other samples showed values within the low normal range.

The initially selected target metabolites tryptamine and 3‐MT were removed from the measurement, since the clinical relevance of tryptamine is questionable and 3‐MT was undetectable in any sample.

For SAM and SAH we could establish norm values out of 690 samples in CSF (Table 12). For plasma, previously published reference values were applied [16].

**TABLE 12:** Reference values for S‐adenosylmethionine (SAM) and S‐adenosylhomocysteine (SAH) in CSF and Plasma: Reference values [nmol/L] for S‐adenosylmethionine (SAM) and S‐adenosylhomocysteine (SAH) in CSF and plasma samples by the measurement and data analyses of 690 CSF samples.

	CSF			Plasma		
	SAM	SAH	SAM/SAH	SAM	SAH	SAM/SAH
0–90 d	171–355	12–41		29.6–118.0	11.8–39.4	
91–183 d	154–323	8–30		29.6–118.0	11.8–39.4	
≥ 184 d	112–254	5–24		29.6–118.0	11.8–39.4	
0–9 y			8–32			1.1–5.1
10–199 y			7–26			1.1–5.1

Abbreviations: d, days (age); SAH, S‐adenosylhomocysteine; SAM, S‐adenosylmethionine; y, years.

After optimization steps, a reliable and dependable method was established in CSF for SAM and SAH. Unfortunately, no positive control CSF sample was available. Under the assumption that changes might be seen due to methylation interaction, 65 patients with dihydropteridinereductase (DHPR) deficiency, Kearns Sayre syndrome, or folate deficiency were analyzed, but no relevant changes were observed.

Since we often did not have CSF samples from patients with a known or suspected methylation disorder, the methodology was extended to plasma. In plasma, SAM and SAH were tested in patients with homocystinuria, in two siblings with methionin‐adenosyltransferase I/III (MAT) deficiency, one patient with ADK and three patients with SAHH deficiency, one patient with MTHFR deficiency under betaine supplementation and one patient with NSUN3 deficiency under SAM supplementation. The patients with SAHH and ADK deficiency showed the expected elevations of SAM and SAH although levels were not as high as described in the literature. The patient under SAM supplementation showed elevated concentrations of SAM as expected. In patients with homocystinuria elevations of SAM and SAH were seen. The 5‐MTHF patient showed elevated concentrations of SAH (Table 10).

In the older MAT sibling, we found both normal values and reduced parameters for SAH. In the younger sibling, SAM and SAH were either normal or slightly elevated, which did not correspond to the results expected in the literature. Unfortunately, no CSF samples were available.

Elevated SAM and SAH concentrations in plasma were found not to be specific for diseases in the area of methylation disorders, as they were often significantly elevated in patients with acute liver problems often going along with an increased methionine. Follow‐up examinations in these patients after improvement of the acute liver disease showed often normalization of SAM, SAH, and methionine.

#### 3.1.2 Second Run: Pterins

Reference values for the determination of neopterin and biopterin measured by LC–MS/MS were established with 202 CSF samples and correlated well with already published reference data. However, in contrast to the established values, our data didn't reveal an age‐related difference (Table 13). To evaluate the method, CSF samples were again taken from patients with known disorders of BH4 metabolism (2 × DHPR, 2 × GTPCH, and 2 × PTPS deficiency). The LC–MS/MS method confirmed the known pathological patterns (Table 6).

**TABLE 13:** Reference values for pterins: Reference ranges [nmol/L] in CSF samples for LC–MS/MS and HPLC‐FLD method determined by the measurement and data analysis of 202 CSF samples.

	LC–MS/MS		
	Neopterin	Biopterin	%B
0–4 y	5–38	5–37	20–76
5–199 y	5–38	5–37	20–76

*Note:* Age: years (y).

#### 3.1.3 Third Run: Amino Acids in CSF

A commercially available kit capable of analyzing 48 amino acids was used to transfer the analysis of 38 amino acids previously performed by IEC to a LC–MS/MS method (MassChrom Amino Acid Analysis, 75 111, Chromsystem, Gräfelfing, Germany).

As the kit has only been approved for measuring amino acids in plasma and urine to date, its use for CSF samples first had to be verified by means of a parallel measurement of 40 CSF samples by the LC–MS/MS and IEC method. Thereby, the results of the IEC method for measurable 33 amino acids were compared to the results determined by the LC–MS/MS method. Homocarnosine has no diagnostic relevance and was therefore not integrated into the method by Chromesystems Kit.

With the new LC–MS/MS method for amino acids, new reference values in CSF samples were established out of 230 control CSF samples (Table 14). All confirmed diagnoses in residual samples (NKH, DHPR deficiency, 3‐phosphoglycerate dehydrogenase (3‐PHGDH) deficiency, glutamine‐synthetase deficiency and asparagine‐synthetase deficiency) were reliably diagnosed, as shown in Table 7. Furthermore, in both patients with known antiquitin deficiency (ATQ), an elevation of pipecolic acid was detected, whereas in all other samples the concentration of pipecolic acid was found within the normal range (Table 7).

**TABLE 14:** Reference values for amino acids: Reference values [μmol/L] in CSF samples for different ages.

AS (μmol/L) in CSF	Age													
	30 d	3 m	6 m	9 m	12 m	2 y	3 y	4 y	5 y	6 y	10 y	15 y	18 y	20 y
1‐Methylhistidine	0–1.3													
Alpha‐aminobutyric acid	0–5.3													
Beta‐aminoisobutyric acid	0–0.1													
3‐Methylhistidine	0–2.5													
4‐Hydroxyproline	0–2.5													
Acetyltyrosine	0–0.1													
Adenosylhomocysteine	0–1													
Alanine	13–36													
Allo‐Isoleucine	0–1													
Alpha‐aminoadipic acid	0–0.2													
Anserine	0–0.2													
Arginine	0–30													
Argininosuccinic acid	0–0.5													
Asparagine	4–11													
Aspartic acid	0–1													
Beta‐alanine	0–0.5													
Carnosine	0–0.3													
Citrulline	0–4													
Cystathionine	0–0.5													
Cysteine sulfate	0–0.2													
Cystine	0–0.3													
Ethanolamine	0–35													
γ‐aminobutyric acid	0–0.6													
Glutamine	300–700													
Glutamic acid	0–1													
Glycine	3–8													
Histidine	5–22													
Homocitrulline	0–0.2													
Homocystine	0–0.2													
Hydroxylysine	0–0.2													
Isoleucine	2–8													
Leucine	5–21													
Lysine	0–30													
Methionine	1–5.5													
Ornithine	0–11													
Phenylalanine	4–14													
Phosphoethanolamine	1–10													
Phosphoserine	0–1													
Pipecolic acid	0–0.2													
Proline	0–2.5													
Saccharopine	0–0.5													
Sarcosine	0–0.2													
Serine	33–68	33–68	32–65	31–63	31–63	28–56	27–52	26–49	25–48	25–48	24–44	24–44	23–42	22–40
Taurine	2–14													
Threonine	13–66	13–64	13–62	13–59	13–56	13–50								
Tryptophan	1.3–5													
Tyrosine	3–25													
Valine	5–27													

*Note:* Determined by the measurement and data analysis of 234 CSF samples measured and calculated by a modification of the Kit of Chromsystems (CS) analytical method.

#### 3.1.4 Fourth Run: Vitamin B_6_ Metabolites in CSF

The method and its norm values were adapted according to the paper of Rossmann et al. [14] (Table 15). In 562 CSF samples with either a known or unknown neurometabolic disease we measured the vitamin B_6_ metabolites. In the four patients with a genetically confirmed vitamin B_6_‐dependent epilepsy we could found decreased values for PLP in 3 patients, one patient was already under supplementation (Table 8). In further 39 patients low values for PLP were seen, often only mildly decreased. Two samples showed other pre‐analytic changes in CSF examinations so that the PLP‐reduction is most likely unspecific due to somewhat less than perfect handling of the samples (e.g., delayed freezing, blood contamination).

**TABLE 15:** Reference values for vitamin B_6_ metabolites: Reference values [nmol/L] in CSF samples for vitamins B_6_ metabolites from Rossmann et al. [14].

	PM	PL	PN	PLP	PA	PLP/PL
0–30 d	0–8	6.7–56	0–5	11–51	0–5	0.5–2.6
31–90 d	0–8	6.7–55	0–5	11–50	0–5	0.5–2.6
91–183 d	0–8	6.7–52	0–5	10–48	0–5	0.5–2.6
184–365 d	0–8	6.7–48	0–5	10–45	0–5	0.5–2.6
1–1.5 y	0–8	6.7–45	0–5	9–43	0–5	0.5–2.6
1.6–2 y	0–8	6.7–42	0–5	9–40	0–5	0.5–2.6
2.1–2.5 y	0–8	6.7–38	0–5	9–39	0–5	0.5–2.6
2.6–3 y	0–8	6.7–36	0–5	8–36	0–5	0.5–2.6
3.1–4 y	0–8	6.7–34	0–5	8–36	0–5	0.5–2.6
4.1–5 y	0–8	6.7–32	0–5	7–34	0–5	0.5–2.6
6–199 y	0–8	6.7–30	0–5	7–34	0–5	0.5–2.6

Abbreviations: d, days (age); PA, pyridoxic acid; PL, pyridoxa; PLP, pyridoxal‐5′‐phosphate; PM, pyridoxamine; PN, pyridoxine; y, years.

In 68 patients, CSF sampling was under vitamin B_6_ supplementation. As expected, treatment with vitamin B_6_ prior to CSF sampling resulted in an increase in some to all vitamers apart from PLP, which remained within the normal range in 82% of cases, even under supplementation.

#### 3.1.5 Fifth Run: Purines and Pyrimidines

The range of purines and pyrimidines was measured in 141 CSF samples and normal values were established (Table 9). The determination of purines and pyrimidines revealed newly diagnosed adenylosuccinate lyase deficiency in one patient, Lesch–Nyhan syndrome in two patients, and molybdenum cofactor type A deficiency in one.

In one patient, non‐specific changes were found that did not lead to a clear diagnosis. At the same time, these changes were also found in the purines/pyrimidines in the urine. Since the follow‐up examination of the urine showed normal results, non‐specific changes can be assumed.

In CSF further non‐specific increases (*n* = 47 samples) could be well distinguished from “true” abnormalities, since the changes were not typical for any known disease. Out of these samples, 20 samples were validated as “normal” and 23 as unspecific without any need to repeat the analysis (in 9 samples we had additionally measurements in urine showing either normal or also mild unspecific changes). Only for four patients a further analysis in urine was recommended; in one patient the diagnosis of molybdenum co factor deficiency could be made.

#### 3.1.6 Sixth Run: Guanidino Compounds

The analysis of guanidino compounds in CSF with LC–MS/MS is already a well‐established measurement method used in metabolic laboratories [4, 12]. The method was integrated in the NeuroMetabolom panel and its efficacy proven by positive controls: 3 patients with GAMT deficiency were reliably detected by the elevation of GAA. Although it is known that patients with a CT transporter deficiency are best detected in urine, we could see an increased CT level in CSF in two patients before treatment and in one patient under treatment (Table 16).

**TABLE 16:** Reference values and confirmation of known deficiencies in creatine metabolism: Reference (Ref.) values [μmol/L] in CSF samples for guanidino compounds method determined by the measurement and data analysis of 127 CSF samples.

	GAMT		GAMT		GAMT		CTD	CTD	CTD
Medication	b.t.	u.t.	b.t.	u.t.	b.t.	u.t.	b.t.	u.t.	b.t.
Sex	f		f		f		m	m	m
Age	10 y 9 m	14 y 1 m	1 y 11 m	2 y 10 m	7 y 10 m	8 y 11 m	14 y 7 m	18 y 6 m	3 y 6 m
Guanidinoacetate (Ref. value 0.01–0.16)	17.12 ↑	6.27 ↑	6.14 ↑	3.66 ↑	8.42 ↑	1.41 ↑	0	0.11	0.46 ↑
Creatine (Ref. value 25–60)	2.3 ↓	15.5	0.3 ↓	18.6	0.5 ↓	54	57.1	85 ↑	92.3 ↑

*Note:* Guanidino compounds concentration in CSF of patients with GAMT (guanidinoacetate methyltransferase deficiency) and CTD (creatine transporter deficiency) determined by LC–MS/MS method. Medication: before treatment (b.t.), under treatment (u.t.). Sex: male (m), female (f). Age: age of sampling, days (d), months (m), years (y).

### 3.2 Clinical Results

Following the successful transfer of the individual analysis platforms to the new LC–MS/MS platform, 86 patients with a known neurometabolic disorder going along with biochemical changes were examined using the newly established NeuroMetabolom Panel. The LC–MS/MS CSF results confirmed the previous diagnosis in all patients based on the typical biochemical pattern.

To verify its applicability in routine clinical practice, the NeuroMetabolom Panel was also used to evaluate 166 CSF samples from patients with suspected neurometabolic disorders. Within this cohort, a new neurometabolic diagnosis could be made in 6 patients (tyrosine hydroxylase deficiency (THD), molybdenum cofactor deficiency, aromatic L‐amino acid decarboxylase deficiency (AADC), GTPCH deficiency, and twice Lesch–Nyhan syndrome) by the LC–MS/MS method.

Some of the remaining samples showed slight changes in individual metabolites, but these can be clearly distinguished from known pathological patterns and are therefore interpreted as non‐specific.

In this cohort a genetic diagnosis could be made in 31 patients with epilepsy, where no biochemical changes are expected. In some of those patients, mild but unspecific changes were observed in the NeuroMetabolom Panel. In a further 8 patients, genetic findings of unclear significance were present.

## 4 Discussion

The aim of this work was to establish a comprehensive, reliable, targeted and rapid measurement method using LC–MS/MS in the form of a neurometabolic panel for implementation in clinical routine. The neurometabolic panel is designed to enable a broad diagnostic coverage using minimal sample material, streamline the workflow for physicians with limited experience in metabolic disorders, and ultimately reduce the time to diagnosis and initiation of therapy.

Due to the wide variation of physical, chemical, and concentration properties of the relevant metabolites, it was necessary to divide the panel into six separate LC–MS/MS runs requiring different sample preparation, turnaround time, and columns to ensure reliable quantitative measurement of all metabolites, regardless of their different biochemical properties (Table 1).

The following conclusions could be drawn from the individual analysis runs:

### 4.1 Various Runs With Different Metabolites

#### 4.1.1 Biogenic Amines, Biogenic Amines Metabolites and Sepiapterin in CSF, SAM, and SAH in CSF and Plasma

Looking at the first run, the biogenic amines and 5‐MTHF results are very comparable to the previously used method. The newly established reference values for the LC–MS/MS methods correlate well with the values given in the literature [17].

Although, as expected, the results of the new values are not identical to those of the previous analytical platforms in terms of numerical values (biogenic amines themselves have some mild fluctuations, but the tendency is always the same), the pathological samples confirm the known biochemical pattern, so that a clear diagnosis can be confidently made with the new method. In addition to diagnosis, the determination of biogenic amines and their metabolites plays a decisive role in the follow‐up of specific therapies and in the assessment of the effectiveness of new innovative therapies. The accurate quantification of metabolites, which has been verified by interlaboratory tests, and the reliable detection of new metabolites like VLA in CSF can therefore be used for the standardized follow‐up in identified patients. One example is the gene therapy for AADC deficiency [18]. Furthermore, it was shown that the measurement of 5‐MTHF is significantly less influenced by hemolytic sample or nonideal pre‐analytics and shows significantly fewer non‐specific reductions (data not shown).

The measurement of tryptamine and 3‐MT was ultimately not included in the panel, as the clinical significance of tryptamine remains highly controversial and was not elevated in any of our samples.

In the determination of 3‐MT, a strong matrix effect of the CSF is most likely to result in undetectable low values, which is contrary to the information in the literature [13]. As the clinical relevance of this parameter appears to be very low, no further emphasis was placed on optimizing the measurement methodology.

Sepiapterin was reliably detected in one patient with SRD, while it remained within the normal range in all other samples. This confirms the reliability of this parameter for diagnosing SRD. However, sepiapterin has so far only been analyzed in extremely rare instances and upon special request in our laboratory. This approach carries the risk of overlooking this extremely rare disorder. The addition of sepiapterin to a routine panel overcomes this limitation and contributes to reliable diagnosis.

The significance of GABA, which has also been newly added to the neurometabolic panel, is less clear. More precise confirmation or assessment of a possible increase would only be possible with a positive control, for example, through CSF from a patient with SSADH or GABA transaminase deficiency. Currently, changes in GABA in CSF have mainly been found in patients with epileptic seizures, psychiatric disorders, and those taking epilepsy medication. Furthermore, it could be shown that GABA increases when the CSF sample is stored at room temperature instead of being stored immediately frozen (data not shown).

Changes in SAM and SAH, intermediate metabolites of the methylation circle, often go along with elevations of methionine and changes in homocysteine, but especially in ADK deficiency and in rare cases in SAHH deficiency, methionine and homocysteine can remain normal, so that the biochemical diagnosis can only be made by measuring SAM and SAH [6]. The method was successfully established in CSF and plasma, and test‐specific parameters and reference values were created. To note is that we focused on elevated values rather than reduced values as typically seen in psychiatric patients [18].

In CSF low values of SAM went along with suboptimal sample handling. Interestingly, some of those samples also had a low HVA. Elevated concentrations were also seen in hemolytic samples as well as in samples having elevated concentrations for 5‐MTHF.

SAH also tends to be elevated in hemolytic samples or in samples in which neopterin is elevated. Further investigations are needed to better assess the significance of SAM/SAH in CSF as a reliable parameter. Furthermore, interpretation is limited since no positive controls have been available so far.

Due to the availability of positive controls in plasma, the method was extended to plasma. As expected, the measured SAM was significantly increased under SAM supplementation. The changes in homocystinuria, 5‐MTHF, and ADK deficiency were also appropriate (although higher values are often given in literature, especially for ADK deficiency), but not in the two siblings with MAT deficiency [6]. In the latter case, one would expect next to a high methionine a normal or reduced concentration of SAM and normal values of SAH, which was not constantly the case in our samples.

As described in literature, SAM and SAH are also elevated in patients with a liver affection and normalized once the clinical symptoms improved [7]. Due to the non‐specific increase in SAM and SAH in liver failure, a biochemical diagnosis of a methylation disorder is not possible, at least not in acute liver failure. This requires follow‐up controls or genetic clarification.

However, since methylation disorders are associated with liver problems, rapid, valid metabolic diagnostics would be especially relevant for these patients.

#### 4.1.2 Pterins in CSF

Looking for neopterin and biopterin concentrations in CSF, the results with the new LC–MS/MS are very good and comparable to the HPLC‐FLD method used up to date in our laboratory method (Table 6). Pterins must be measured in a separate run within the LC–MS/MS method since oxidation with MnO_2_ is required. During this oxidation step, other analytes are also oxidized and can be degraded, and thus cannot be measured in parallel with the pterins [19].

#### 4.1.3 Amino Acids in CSF

The determination of amino acids in CSF is of great importance in clinical practice. It is one of the most frequently requested tests in CSF, especially for ruling out NKH or serine biosynthesis disorders.

The newly established LC–MS/MS method for CSF using the Chromsystems kit for plasma analysis procedure and reagents together with a modified procedure of the standards and quality controls provided reliable values. All confirmed diagnoses in residual samples were diagnosed, and the pre‐existing panel on measurable amino acids can now be extended, although not all of them are of clinical interest. It is particularly worth mentioning that the isomeric amino acids allo‐isoleucine, isoleucine and leucine, relevant for the diagnosis of MSUD, are now integrated into this one amino acid method and can be chromatographically separated prior to MS/MS and quantified subsequently. With our previous measurement methods, a secondary run for this separation was required and therefore led to a delay in confirmation or exclusion of MSUD.

As typical for amino acids, unspecific changes can be seen; therefore, interpretation together with the clinical information and pattern recognition remains essential. GABA can also be determined using this method, but as elaborated earlier, there are still limitations in its interpretation and further studies are needed.

Similar to sepiapterin mentioned above, pipecolic acid also had to be requested separately and individually in our laboratory. This parameter is now also integrated into the routine panel and determined together with the amino acids in CSF. The method evaluation showed that an increase in pipecolic acid was detectable in the two known patients with antiquitin deficiency, while pipecolic acid remained within the normal range in all other samples (two further patients with antiquitin deficiency were unfortunately not tested since no material was available anymore). However, it should be noted that pipecolic acid can also be elevated in other diseases (e.g., peroxisomal disorders) and should therefore be supplemented by the determination of pipecolic acid and AASA in blood or urine if clinically suspected [20]. Those parameters can already be measured in urine and plasma by LC–MS/MS, so that it could be integrated in an LC MS/MS plasma and urine panel once it is established.

For everyday clinical use, the rapid measurement and the very small amount of total 25 μL sample material in comparison to the previously necessary 100 μL sample material for the analytical setup (Tables 1, 2, 3) required are particularly noteworthy. After calibration, measurement results of individual tests are possible within 20 min.

#### 4.1.4 Vitamin B_6_ Metabolites in CSF

Since the publication of the initially developed methodology, the determination of vitamin B_6_ metabolites has been offered in our laboratory. Unfortunately, many samples were only taken after the initiation of vitamin B_6_ supplementation. Under supplementation, however, PLP can be normal again and the interpretation of the result is no longer reliable, so that no statement regarding vitamin B_6_‐dependent epilepsy is possible. It must therefore be made clearer in future that the sample must be taken before vitamin B_6_ is administered, although this will certainly not always be possible in everyday clinical practice for logistical reasons. Therefore, a trial vitamin B_6_ test and subsequent genetic testing will always have its place [21].

Nevertheless, the targeted determination of PLP in the CSF can be a rapid diagnostic method and, in our opinion, should always be carried out as standard in children with a seizure disorder, especially in young infants. In our patient collective, we could clearly detect the patients prior to supplementation with a genetically confirmed diagnosis, but a slightly reduced PLP was also found in approx. 10% of all cases. In order not to overlook any genetically confirmed patients, it is not possible to lower the lower limit. Instead we recommend close communication with the treating colleagues in order to be able to interpret the findings in the clinical context.

It is known that PLP is very unstable and can be rapidly degraded by light and delayed freezing [22]. Therefore, also in our cohort, some of the reductions are certainly due to pre‐analytical changes. Further factors like alimentation, medication, or increased consumption in the context of seizures could also be an explanation for decreases and must be observed in more detail. Unfortunately, due to the nature of this study, no or incomplete clinical information was available. It can, therefore, not completely be excluded that among the CSF samples with supposedly non‐specific reductions, cases with genetically determined vitamin B_6_‐dependent epilepsy were included. In addition to the determination of PLP in CSF, the determination of 2S,6S‐2S,6R‐oxopropylpiperidine‐2‐carboxylic acid (2‐OPP), 6‐oxopiperidine 2‐carboxylic acid (6‐oxo‐pip), and PM, and especially its ratio in plasma is also playing an increasingly important role according to the literature and the experiences of our and other laboratories [23, 24, 25]. Establishing a further measurement method of vitamin B_6_ metabolites in plasma with the metabolites mentioned above using LC–MS/MS is in progress.

#### 4.1.5 Purines and Pyrimidines in CSF

Purine and pyrimidine disorders are very rare and can only be diagnosed in urine in specialized metabolic centers [26]. Due to the clinical rarity, broad clinical symptoms and the very specific request, diagnosis is often delayed and often only reached by genetic tests [27].

To our knowledge and according to the literature, purines and pyrimidines have not yet been examined or measured in CSF [28]. At the start of the study, it was therefore unclear whether the expected changes in affected patients would be detectable in CSF. However, the expected changes were found in all four of our pathological samples with confirmed diagnoses. We therefore assume that relevant changes can be seen in the CSF and that the additional determination of purines and pyrimidines in the CSF is a good addition for at least a part of the diseases.

It should be noted that in some other samples isolated metabolites were mildly increased. But those changes did not show any typical pattern of a known disease and could be interpreted as “unspecific result”. Even though the determination of purines and pyrimidines in urine is likely to remain the gold standard, we consider the integration of purines and pyrimidines in the CSF panel to be a major advantage, especially since the determination of purines and pyrimidines is often not explicitly requested in clinical practice, as we have seen in one of the patients described in this paper (molybdenum co‐factor deficiency).

#### 4.1.6 Guanidino Compounds in CSF

The determination of guanidino compounds has been an existing measurement method using LC–MS/MS for years and was simply integrated into the new panel [4, 12]. Because of highly different concentration levels of CT compared to the other analytes, the measurement of GAA and CT remained as a single run and was not integrated in one of the other runs described here.

Patients with GAMT deficiency were reliably diagnosed in CSF. However, it is known that patients with a CT transporter deficiency are best detected in urine; hence, CSF diagnostics will not be able to replace urine diagnostics. In our three patients, we could though see an increased CT level (two patients before treatment, one under treatment) so that diagnostics in CSF might be a supportive tool. The very rare AGAT deficiency won't be detected in our NeuroMetabolom panel since such low values cannot be detected in CSF.

### 4.2 Challenges

#### 4.2.1 Preanalytics

Compared to the previous CSF measurement method, the LC–MS/MS method appears to be less sensitive to preanalytical changes. Of particular note here is 5‐MTHF, which has been shown to be more stable, especially in hemolytic samples (data not shown). Nevertheless, correct preanalytics should also be ensured when using the LC–MS/MS method. The CSF sample should be frozen immediately after collection, protected from light, and shipped frozen. If it is contaminated with blood, it should first be centrifuged and then shipped frozen. This is particularly true for vitamin B_6_ metabolites and SAM/SAH.

In everyday clinical practice, however, even suboptimally collected samples can often be interpreted in the overall context, and in most cases only a few samples cannot be conclusively interpreted due to faulty preanalytics.

#### 4.2.2 Study Design

In this study, clinical data were primarily obtained from the written request forms available to us. In most cases, it was not possible to follow up on the clinical course or to perform follow‐up examinations. We also often did not have access to precise information regarding medication intake as a potential confounder, pre‐analytical changes due to factors such as bloody contamination or delayed freezing, or diagnoses made after the samples were submitted. Clear elevations due to supplementation of SAM, CT or Vitamin B6 could be reliably seen and those samples were excluded from norm value calculation.

### 4.3 The NeuroMetabolom Panel From Clinical Point of View

With the new panel, it is now possible to perform comprehensive neurometabolic diagnostics from just one CSF sample using a single measurement platform. A new addition is the determination of vitamin B_6_ metabolites, pipecolic acid, purines and pyrimidines, and sepiapterin in routine diagnostics in CSF.

The determined reference values are consistent with those from previously used methods and the literature, apart from the newly established amino acids where we could see mild differences compared to published reference values. For each run and its related diseases, it was shown by means of pathological samples that these can be detected validly using the new measurement method except for SAM and SAH in CSF and for GABA, since no positive control was available.

From our point of view, the request for a metabolic panel must be the future standard, as the clinical assignment of symptoms to a specific disease is hardly possible [29]. Since many neurometabolic diseases are associated with changes in the CSF and its collection remains an invasive procedure with corresponding potential risks, a panel for CSF diagnostics is of great importance [30]. The use of LC–MS/MS lends itself to this, as this platform enables the determination of many metabolites in a short time [31]. A great advantage is the very little sample volume required for this platform and the shorter run time.

However, it must be kept in mind that this technology requires the preparation and measurement of calibration series and that several standards must be tested regularly. Therefore, this method is rather time‐consuming for a small number of samples, whereas for larger numbers of samples the time for the preparation of the calibration series and the measurement of the standards is shorter than in other analytical methods, and the rapid turnaround time of the individual sample is advantageous.

The switch to a single measurement method reduces the variety of devices and thus the logistical effort in terms of device maintenance, evaluation of findings and staff training. This means that significantly more experience is gained in the LC–MS/MS methodology and thus sources of error are reduced. Furthermore, thawing cycles can be reduced so that optimum sample measurement is possible and fewer unspecific changes occur due to suboptimal sample logistics.

In addition, the smaller number of devices means that more laboratory space is available and maintenance costs can be reduced. Even though the high acquisition costs of MS/MS devices must be taken into account, we assume that costs will fall in the future due to widespread use.

NeuroMetabolom focuses on measuring CSF, as many neurometabolic disorders lead to cerebral impairments with specific metabolite constellations. However, the LC–MS/MS methodology is not limited to CSF and is now already also used in various measurements (including amino acids, guanidino compounds, pipecolic acid, purines/pyrimidines, SAM/SAH) in urine and/or blood/EDTA plasma. Furthermore, this method allows integration of new metabolites enabling an expansion of the panel, which should be a future aim.

## 5 Conclusion

With the new LC–MS/MS method, it is now possible to detect reliably a wide range of relevant neuro‐metabolic diseases by one analytical platform. In addition to neurotransmitter diseases and disorders in folate metabolism, vitamin B_6_‐dependent epilepsies and disorders in guanidino and amino acid metabolism can also be diagnosed.

In our opinion, despite significant advances in genetics, metabolic diagnostics will continue to play a central role in everyday clinical practice in the future. This is because, unlike genetics, it reflects actual metabolic changes in the body and therefore might help to distinguish the severity of affection or to distinguish whether the patient is affected or merely a carrier. It furthermore allows disease and therapeutic monitoring over time. In summary, the new LC–MS/MS method is reliable and fast, requires significantly less CSF, optimizes sample handling, reduces the likelihood of errors, and simplifies staff training by reducing the number of measurement methods to just one. It also offers the possibility of integrating additional metabolites over time or extending the measurement method to blood and urine. Furthermore, it allows comprehensive diagnostics in neuropediatrics and significantly shortens the time to diagnosis as well as a possible treatment start.

Therefore, in our opinion, LC–MS/MS and a NeuroMetabolom approach will be—next to genetic testing—the future in metabolic diagnosis and will replace the established step‐by‐step diagnostic.

## Author Contributions

Stine Christ and Thomas Opladen drafted the manuscript and were responsible for the collection and medical evaluation of patient samples. Sylvia Richter, Julia Rossmann, and Glynis Klinke were responsible for establishing and optimizing the method from a technical point of view, measurement, and evaluation of the samples. Jürgen Günther Okun and Nenad Blau supervised the project from a laboratory point of view. Sven Garbade supported the team in establishing and calculating the reference values. Georg Friedrich Hoffmann initiated the project and enabled the initial foundation. All authors revised the manuscript and approved the submission.

## Funding

The study was further supported by the Dietmar Hopp Foundation, Leon‐Rot, Germany (23011222) (project “Newborn screening 2020” to G.F.H.).

## Ethics Statement

All procedures followed were in accordance with the ethical standards of the responsible committee on human experimentation (institutional and national) and with the Helsinki Declaration of 1975, as revised in 2013. The study was approved by the Institutional Ethics Committee of the University of Heidelberg (S‐471/2014) and by all contributing clinical partners, and listed in the German Clinical Trials Register, https://www.drks.de.

## Consent

Informed consent was obtained from all patients or their legal guardians prior to being included in the study.

## Conflicts of Interest

The authors declare no conflicts of interest.

## Supporting information


**Data S1:** jimd70167‐sup‐0001‐Supinfo1.docx.

## Data Availability

The data that support the findings of this study are available from the corresponding author upon reasonable request.
