# Supplementary material for: A Novel Multimodal LC–MS/MS Panel for the Comprehensive Diagnosis of Neurometabolic Disorders in CSF
Source: J Inherit Metab Dis. 2026 Apr 6;49(3):e70167. doi: 10.1002/jimd.70167 (PMC13053135; doi:10.1002/jimd.70167)
Supplement: Supplementary file 1 — Data S1: jimd70167‐sup‐0001‐Supinfo1.docx. [file JIMD-49-0-s001.docx]

**Supplementary data:**

Table S 1: Transitions and MS parameters of the LC-MS/MS methods first, second and sixth run. Instrument: Waters Xevo TQ-S.

| **Run** | **Tune page settings** | **Analyte** | **IS** | **Quantifier/ Qualifier** | **ESI** | **MRM** | **Dwell­time** | **Cone voltage** | **Collision energy** | **time scan** |
| --- | --- | --- | --- | --- | --- | --- | --- | --- | --- | --- |
|  |  |  |  |  |  | **m/z** | **/ ms** | **/ V** | **/ V** | **/min** |
| **First run** | Capillary (kV) 2.50 Source Temperature (°C) 150 Desolvation Temperature (°C) 550 Cone Gas Flow (L/Hr) 180  Desolvation Gas Flow (L/Hr) 1000 Collision Gas Flow (mL/Min) 0.14 Nebuliser Gas Flow (Bar) 6.00 | 13C5-5MTHF | - | - | + | 460.1 > 313.1 | 15 | 34 | 20 | 4.4 – 5.7 |
|  |  | 3-OMD D_3_ | - | - | + | 215.1 > 156.1 | 15 | 2 | 14 | 4.0 – 5.3 |
|  |  | 5-HIAA D_6_ | - | - | + | 198.0 > 151.0 | 30 | 30 | 14 | 6.0 – 7.5 |
|  |  | 5-HTP D_3_ | - | - | + | 223.9 > 137.0 | 15 | 28 | 24 | 4.2 – 5.6 |
|  |  | HVA D_5_ | - | - | - | 186.1 > 142.1 | 40 | 18 | 8 | 6.4 – 7.8 |
|  |  | L-Dopa D_3_ | - | - | + | 201.1 > 154.3 | 15 | 20 | 13 | 3.3 – 4.5 |
|  |  | PN D_2_ | - | - | + | 172.2 > 136.2 | 15 | 66 | 20 | 3.2 – 4.5 |
|  |  | SAH D_4_ | - | - | + | 389.1 > 136.0 | 15 | 8 | 40 | 3.3 – 4.4 |
|  |  | SAM D_3_ | - | - | + | 402.1 > 249.9 | 70 | 8 | 14 | 1.0 – 2.5 |
|  |  | 3-OMD | 3-OMD D_3_ | Quant | + | 212.2 > 195.1 | 15 | 24 | 8 | 4.0 – 5.3 |
|  |  |  |  | Qual |  | 212.2 > 153.1 | 15 | 24 | 14 |  |
|  |  | 5-HIAA | 5-HIAA D_6_ | Quant | + | 192.0 > 146.0 | 30 | 34 | 26 | 6.0 – 7.5 |
|  |  |  |  | Qual |  | 192.0 > 91.0 | 30 | 34 | 32 |  |
|  |  | 5-HTP | 5-HTP D_3_ | Quant | + | 220.9 > 162.0 | 15 | 28 | 18 | 4.2 – 5.6 |
|  |  |  |  | Qual |  | 220.6 > 134.0 | 15 | 28 | 14 |  |
|  |  | 5-MTHF | 13C5-5MTHF | Quant | + | 460.1 > 313.1 | 15 | 20 | 20 | 4.4 – 5.7 |
|  |  |  |  | Qual |  | 460.1 > 180.2 | 15 | 20 | 20 |  |
|  |  | GABA | SAM D_3_ | Quant | + | 104.1 > 87.1 | 25 | 13 | 11 | 1.0 – 2.5 |
|  |  |  |  | Qual |  | 104.1 > 69.1 | 25 | 13 | 10 |  |
|  |  | HVA | HVA D_5_ | Quant | - | 181.1 > 122.1 | 40 | 2 | 14 | 6.4 – 7.8 |
|  |  |  |  | Qual |  | 180.8 > 121.9 | 40 | 6 | 16 |  |
|  |  | L-Dopa | L-Dopa D_3_ | Quant | + | 198.1 > 107.0 | 15 | 20 | 24 | 3.3 – 4.5 |
|  |  |  |  | Qual |  | 198.1 > 181.1 | 15 | 20 | 8 |  |
|  |  | SAH | SAH D_4_ | Quant | + | 385.1 > 133.6 | 15 | 14 | 22 | 3.3 – 4.4 |
|  |  |  |  | Qual |  | 385.1 > 87.9 | 15 | 14 | 38 |  |
|  |  | SAM | SAM D_3_ | Quant | + | 399.1 > 250.0 | 70 | 22 | 16 | 1.0 – 2.5 |
|  |  |  |  | Qual |  | 399.1 > 96.9 | 70 | 22 | 32 |  |
|  |  | Sep | PN D_2_ | Quant | + | 238.0 > 192.0 | 15 | 8 | 16 | 4.4 – 5.3 |
|  |  |  |  | Qual |  | 238.0 > 164.0 | 15 | 8 | 22 |  |
|  |  | VLA | PN D_2_ | Quant | - | 210.98 > 133.99 | 15 | 30 | 11 | 5.6 – 6.8 |
|  |  |  |  | Qual |  | 210.98 > 149.98 | 15 | 60 | 10 |  |
| **Second run** | Capillary (kV) 0.7 Source Temperature (°C) 150 Desolvation Temperature (°C) 600 Cone Gas Flow (L/Hr) 180 Desolvation Gas Flow (L/Hr) 900 Collision Gas Flow (mL/Min) 0.15 Nebuliser Gas Flow (Bar) 6.00 | 13C5Neopterin | - |  | + | 259.2 > 194.2 | 25 | 12 | 20 | 1.5 - 3.0 |
|  |  | Biopterin D_3_ | - |  | + | 241.1 > 180.1 | 25 | 30 | 25 | 3.0 - 5.0 |
|  |  | Neopterin | 13C5Neopterin | Quant | + | 254.2 > 206.0 | 40 | 28 | 18 | 1.5 - 3.0 |
|  |  |  |  | Qual |  | 254.2 > 236.0 | 40 | 28 | 14 |  |
|  |  | Biopterin | Biopterin D_3_ | Quant | + | 238.1 > 178.1 | 40 | 30 | 18 | 3.0 - 5.0 |
|  |  |  |  | Qual |  | 238.1 > 194.1 | 40 | 30 | 22 |  |
| **Sixth run** | Capillary (kV) 0.7 Source Temperature (°C) 150 Desolvation Temperature (°C) 600 Cone Gas Flow (L/Hr) 180 Desolvation Gas Flow (L/Hr) 900 Collision Gas Flow (mL/Min) 0.15 Nebuliser Gas Flow (Bar) 6.00 | Creatine D_3_ (CT-IS) | - | - | + | 135.1 > 47.1 | 10 | 60 | 16 | 2.0 – 4.0 |
|  |  | Guanidinoacetic acid D_2_ (GAA-IS) | - | - | + | 120.1 > 78.1 | 10 | 20 | 11 | 1.6 – 3.0 |
|  |  | Creatine (CT) | CT-IS | Quant | + | 132.1 > 44.1 | 10 | 60 | 16 | 2.0 – 4.0 |
|  |  |  |  | Qual |  | 132.1 > 90.1 | 10 | 60 | 12 |  |
|  |  | Gunidinoacetic acid (GAA) | GAA-IS | Quant | + | 118.1 > 76.1 | 10 | 20 | 11 | 1.6 – 3.0 |
|  |  |  |  | Qual |  | 118.1 > 101.1 | 10 | 20 | 10 |  |

Table S 2: Supplementary Data precision, accuracy, sensitivity and linearity of the methods first, second and sixth run.

| **Run** | **LC conditions** |  | **Rt (min)** | **Working range** | **Linearität** | **LLOD** | **LLOQ** | **Interday** | | | | **Intraday** | | | |
| --- | --- | --- | --- | --- | --- | --- | --- | --- | --- | --- | --- | --- | --- | --- | --- |
|  |  |  |  |  |  |  |  | n = 10 | | | | n = 10, 6th run n = 6 | | | |
|  |  |  |  |  |  |  |  |  | Conc. | RSD (%) | AC (%) |  | Conc. | RSD (%) | AC (%) |
| **First run "Neurotransmitter"** | Column ACE Excel C18-PFP (100x3mm, 1.7µm) 25°C   Mobile phases 0.2 % formic acid and acetonitrile  flow 0.3 L/min runtime 12,5 min | 5-MTHF* | 5.07 | 0 - 500 nM | 0.99815 | 2 nM | 6 nM | QC low | 27.7 nM | 4 | 92 | CSF | 43 nM | 1.2 | 103 |
|  |  |  |  |  |  |  |  | QC med | 83.3 nM | 4.6 | 100 | CSF | 93 nM | 1.5 | 104 |
|  |  |  |  |  |  |  |  | QC high | 250 nM | 6.4 | 101 |  |  |  |  |
|  |  | HVA | 7.2 | 0 - 2000 nM | 0.99787 | 8 nM | 25 nM | QC low | 62.5 nM | 10.3 | 102 | QC low | 62.5 nM | 12.1 | 91 |
|  |  |  |  |  |  |  |  | QC med | 333 nM | 5.0 | 104 | QC med | 333 nM | 6.5 | 106 |
|  |  |  |  |  |  |  |  | QC high | 1000 nM | 6.7 | 105 | QC high | 1000 nM | 5.5 | 104 |
|  |  | 5-HIAA | 6.95 | 0 - 2000 nM | 0.99742 | 8 nM | 25 nM | QC low | 62.5 nM | 5.9 | 107 | QC low | 62.5 nM | 9.5 | 101 |
|  |  |  |  |  |  |  |  | QC med | 333 nM | 5.9 | 101 | QC med | 333 nM | 3.9 | 101 |
|  |  |  |  |  |  |  |  | QC high | 1000 nM | 4.6 | 103 | QC high | 1000 nM | 4.5 | 99 |
|  |  | 3-OMD | 4.71 | 0 - 2000 nM | 0.99756 | 2 nM | 8 nM | QC low | 62.5 nM | 4.8 | 105 | QC low | 62.5 nM | 2.0 | 99 |
|  |  |  |  |  |  |  |  | QC med | 333 nM | 3.4 | 104 | QC med | 333 nM | 2.7 | 107 |
|  |  |  |  |  |  |  |  | QC high | 1000 nM | 3.6 | 103 | QC high | 1000 nM | 0.9 | 108 |
|  |  | 5-HTP | 5.00 | 0 - 500 nM | 0.99578 | 2 nM | 6 nM | QC low | 27.7 nM | 11.5 | 100 | QC low | 27.7 nM | 23.4 | 76 |
|  |  |  |  |  |  |  |  | QC med | 83.3 nM | 7.2 | 100 | QC med | 83.3 nM | 17.1 | 85 |
|  |  |  |  |  |  |  |  | QC high | 250 nM | 5.2 | 102 | QC high | 250 nM | 5.9 | 97 |
|  |  | L-Dopa | 3.99 | 0 - 500 nM | 0.99825 | 1 nM | 2 nM | QC low | 27.7 nM | 5.5 | 99 | QC low | 27.7 nM | 4.6 | 99 |
|  |  |  |  |  |  |  |  | QC med | 83.3 nM | 4.2 | 103 | QC med | 83.3 nM | 2.4 | 102 |
|  |  |  |  |  |  |  |  | QC high | 250 nM | 3.6 | 103 | QC high | 250 nM | 1.9 | 103 |
|  |  | VLA* | 6.39 | 0 - 500 nM | 0.99864 | 2 nM | 6 nM | QC low | 27.7 nM | 9.6 | 104 | CSF | 10 nM | 5.7 | 103 |
|  |  |  |  |  |  |  |  | QC med | 83.3 nM | 6.1 | 103 | CSF | 60 nM | 5.0 | 103 |
|  |  |  |  |  |  |  |  | QC high | 250 nM | 5.1 | 103 |  |  |  |  |
|  |  | GABA | 1.77 | 0 - 2000 nM | 0.9972 | 2 nM | 8 nM | QC low | 88.9 nM | 5.4 | 111 | QC low | 88.9 nM | 3.3 | 107 |
|  |  |  |  |  |  |  |  | QC med | 266.7 nM | 3.0 | 109 | QC med | 266.7 nM | 4.0 | 107 |
|  |  |  |  |  |  |  |  | QC high | 800 nM | 4.1 | 109 | QC high | 800 nM | 2.7 | 108 |
|  |  | Sep* | 4.94 | 0 - 500 nM | 0.98514 | 1 nM | 2 nM | QC low | 27.7 nM | 23.4 | 65 | CSF | 50 nM | 1.6 | 107 |
|  |  |  |  |  |  |  |  | QC med | 83.3 nM | 17.2 | 80 |  |  |  |  |
|  |  |  |  |  |  |  |  | QC high | 250 nM | 7.7 | 97 |  |  |  |  |
|  |  | SAM | 1.68 | 0 - 500 nM | 0.99506 | 1 nM | 2 nM | QC low | 27.7 nM | 4.3 | 111 | QC low | 27.7 nM | 1.0 | 113 |
|  |  |  |  |  |  |  |  | QC med | 83.3 nM | 3.1 | 110 | QC med | 83.3 nM | 1.6 | 112 |
|  |  |  |  |  |  |  |  | QC high | 250 nM | 4.9 | 106 | QC high | 250 nM | 1.3 | 111 |
|  |  | SAH | 3.92 | 0 - 500 nM | 0.99812 | 2 nM | 6 nM | QC low | 27.7 nM | 5.5 | 103 | QC low | 27.7 nM | 6.6 | 115 |
|  |  |  |  |  |  |  |  | QC med | 83.3 nM | 2.8 | 104 | QC med | 83.3 nM | 4.6 | 111 |
|  |  |  |  |  |  |  |  | QC high | 250 nM | 4.5 | 105 | QC high | 250 nM | 3.5 | 113 |
| **Second run Pterins** | Column ACE Excel C18-PFP (100x3mm, 1.7µm) 25°C  mobile phases 0.2 % formic acid and acetonitrile flow 0.3 mL/min runtime 8 min | Neo | 2.39 | 0 - 200 nM | 0.99824 | 0.5 nM | 1.5 nM | QC low | 20 nM | 6.4 | 99 | QC low | 20 nM | 3.5 | 102 |
|  |  |  |  |  |  |  |  | QC med | 74 nM | 5.4 | 99 | QC med | 74 nM | 2.1 | 97 |
|  |  |  |  |  |  |  |  | QC high | 126 nM | 7.5 | 100 | QC high | 126 nM | 1.8 | 97 |
|  |  | Bio | 3.86 | 0 - 100 nM | 0.99819 | 0.5 nM | 1.5 nM | QC low | 7 nM | 4.0 | 107 | QC low | 7 nM | 4.1 | 112 |
|  |  |  |  |  |  |  |  | QC med | 34 nM | 8.2 | 106 | QC med | 34 nM | 3.0 | 105 |
|  |  |  |  |  |  |  |  | QC high | 60 nM | 7.5 | 109 | QC high | 60 nM | 4.2 | 106 |
|  | Column Waters SPERISORB 5µm ODS1 (4.6x250mm) 35°C mobile phase 1 mM KH2PO4, 4 % MeOH flow 1 mL/min runtime 15 min | Neo | 5.55 | 0 - 500 nM | - | 0.1 nM | 1 nM | QC low | 10 nM | 8.2 | 106 | QC low | 10 nM | 6.2 | 111 |
|  |  |  |  |  |  |  |  | QC high | 50 nM | 8.8 | 94 | QC high | 50 nM | 4.9 | 104 |
|  |  | Bio | 9.82 | 0 - 500 nM | - | 0.1 nM | 1 nM | QC low | 10 nM | 9.5 | 100 | QC low | 10 nM | 5.4 | 95 |
|  |  |  |  |  |  |  |  | QC high | 50 nM | 7.2 | 96.5 | QC high | 50 nM | 3.8 | 103 |
| **Sixth run Guanidino compounnds** | Column ACE Excel C18-AR (100x3mm, 1.7µm), 35°C mobile phase 0.4 % formic acid flow 0.2 mL/min runtime 5 min | GAA | 2.61 | 0 - 4,8 µM | 0.9986 | 0.1 µM | 0.25 µM | QC low | 0.75 µM | 4.1 | 93.3 | QC low | 0.75 µM | 2.5 | 92.5 |
|  |  |  |  |  |  |  |  | QC high | 1.5 µM | 3.0 | 93.3 | QC high | 1.5 µM | 3.1 | 94.6 |
|  |  | CT | 2.93 | 0 - 192 µM | 0.9986 | 0.1 µM | 0.25 µM | QC low | 30 µM | 3.9 | 93.4 | QC low | 30 µM | 2.8 | 94 |
|  |  |  |  |  |  |  |  | QC high | 60 µM | 3.0 | 93.5 | QC high | 60 µM | 2.9 | 93.4 |

* Due to low autosampler stability in Ringer's solution, the intraday in CSF was repeated for 5-MTHF, VLA and Sep

Table S 3: Intraday precision (relative standard deviation, RSD (%)) and accuracy (AC (%)) for amino acids determined by 10-fold diluted quality controls (QC) 1, 2 and 3. Concentration (Conc., µmol/L), mean concentration (mean),

| **Intraday (n=6)** | **QC1** | | | | **QC2** | | | | **QC3** | | | |
| --- | --- | --- | --- | --- | --- | --- | --- | --- | --- | --- | --- | --- |
| Amino acid | µmol/L | mean | AC | RSD | µmol/L | mean | AC | RSD | µmol/L | mean | AC | RSD |
| 1-Methylhistidine | 0,433 | 0,422 | 97,4 | 8,0 | 1,13 | 1,09 | 96,6 | 2,4 | 1,54 | 1,51 | 98,2 | 4,3 |
| α-Aminobutyric acid | 0,924 | 0,917 | 99,2 | 5,9 | 4,96 | 4,99 | 100,5 | 3,7 | 8,4 | 8,55 | 101,7 | 3,7 |
| β-Aminoisobutyric acid | 0,775 | 0,747 | 96,4 | 7,1 | 2,63 | 2,57 | 97,5 | 4,8 | 3,96 | 3,93 | 99,3 | 3,8 |
| 3-Methylhistidine | 1,17 | 1,26 | 107,4 | 5,3 | 5,24 | 5,38 | 102,7 | 3,6 | 8,51 | 8,92 | 104,9 | 5,1 |
| 4-Hydroxyproline | 1,47 | 1,43 | 97,0 | 6,9 | 9,07 | 8,92 | 98,3 | 4,4 | 15,8 | 15,56 | 98,5 | 4,0 |
| Acetyltyrosine | 1,05 | 1,01 | 96,3 | 6,6 | 4,66 | 4,60 | 98,8 | 5,1 | 7,35 | 7,43 | 101,0 | 5,5 |
| Adenosylhomocysteine | 0,723 | 0,704 | 97,3 | 8,1 | 2,57 | 2,56 | 99,6 | 3,1 | 3,8 | 3,71 | 97,6 | 2,8 |
| Alanine | 27,3 | 26,19 | 95,9 | 7,2 | 77,5 | 74,9 | 96,7 | 3,7 | 110,9 | 108,3 | 97,6 | 3,7 |
| Allo-Isoleucine | 1,3 | 1,28 | 98,5 | 6,2 | 8,61 | 8,74 | 101,5 | 3,8 | 14,8 | 15,00 | 101,3 | 3,6 |
| α-Aminoadipic acid | 0,532 | 0,524 | 98,5 | 6,0 | 1,3 | 1,29 | 99,5 | 3,4 | 1,82 | 1,83 | 100,7 | 4,1 |
| Anserine | 0,45 | 0,45 | 99,1 | 7,2 | 1,09 | 1,10 | 101,2 | 5,4 | 1,54 | 1,54 | 100,1 | 4,3 |
| Arginine | 4,91 | 4,75 | 96,8 | 5,1 | 18,9 | 18,9 | 100,1 | 2,5 | 28,3 | 28,7 | 101,3 | 2,3 |
| Argininosuccinic acid | 1,17 | 1,25 | 107,0 | 6,9 | 7,75 | 7,78 | 100,4 | 3,8 | 13 | 13,47 | 103,6 | 6,0 |
| Asparagine | 5,75 | 5,55 | 96,6 | 6,6 | 16 | 15,9 | 99,5 | 4,1 | 22,5 | 22,5 | 99,9 | 4,1 |
| Aspartic acid | 3,94 | 4,05 | 102,8 | 8,6 | 14,5 | 14,6 | 100,6 | 4,6 | 22,6 | 23,0 | 101,6 | 3,7 |
| β-Alanine | 1,98 | 1,90 | 95,8 | 8,4 | 6,25 | 6,31 | 100,9 | 5,5 | 9,29 | 9,43 | 101,6 | 3,0 |
| Carnosine | 0,858 | 0,876 | 102,0 | 5,5 | 2,56 | 2,59 | 101,0 | 4,4 | 3,87 | 3,71 | 96,0 | 2,8 |
| Citrulline | 2,28 | 2,23 | 97,8 | 5,9 | 10,8 | 10,8 | 100,3 | 6,5 | 18 | 17,5 | 97,2 | 6,1 |
| Cystathionine | 0,855 | 0,878 | 102,7 | 5,3 | 2,89 | 2,83 | 97,9 | 3,7 | 4,38 | 4,33 | 98,9 | 4,9 |
| Cysteine sulphate | 2,04 | 1,93 | 94,6 | 6,2 | 4,27 | 4,12 | 96,6 | 3,1 | 5,32 | 5,23 | 98,3 | 3,9 |
| Cystine | 1,14 | 1,14 | 100,4 | 4,6 | 7,7 | 7,59 | 98,5 | 4,8 | 13,5 | 13,50 | 100,0 | 2,7 |
| Ethanolamine | 1,65 | 1,58 | 95,5 | 8,1 | 11,5 | 11,5 | 100,0 | 5,4 | 20,3 | 20,6 | 101,6 | 3,8 |
| γ-Aminobutyric acid | 0,399 | 0,393 | 98,4 | 7,1 | 0,999 | 1,007 | 100,8 | 3,8 | 1,44 | 1,438 | 99,9 | 4,3 |
| Glutamine | 52,6 | 51,02 | 97,0 | 7,7 | 123,2 | 120,4 | 97,7 | 3,7 | 157,4 | 154,5 | 98,1 | 3,4 |
| Glutamic acid | 9,65 | 9,54 | 98,8 | 6,3 | 41,9 | 42,7 | 102,0 | 3,7 | 67,9 | 69,3 | 102,0 | 4,2 |
| Glycine | 33,2 | 31,7 | 95,6 | 6,5 | 84,3 | 82,6 | 98,0 | 3,4 | 114,7 | 113,0 | 98,5 | 2,7 |
| Histidine | 6,57 | 6,57 | 100,0 | 7,5 | 18,8 | 19,5 | 103,5 | 4,8 | 26,7 | 27,6 | 103,2 | 3,1 |
| Homocitrulline | 1,5 | 1,36 | 90,4 | 8,5 | 3,7 | 3,52 | 95,3 | 5,3 | 5,01 | 4,84 | 96,6 | 5,9 |
| Homocystine | 0,604 | 0,590 | 97,6 | 7,0 | 1,71 | 1,65 | 96,4 | 4,8 | 2,48 | 2,35 | 94,9 | 5,6 |
| Hydroxylysine | 0,721 | 0,777 | 107,7 | 6,9 | 2,13 | 2,140 | 100,5 | 3,2 | 3,08 | 3,104 | 100,8 | 4,5 |
| Isoleucine | 4,53 | 4,39 | 96,9 | 6,2 | 19,6 | 19,5 | 99,7 | 3,7 | 31,7 | 32,1 | 101,2 | 3,6 |
| Leucine | 11,2 | 11,73 | 104,7 | 5,9 | 37 | 38,5 | 104,0 | 4,0 | 54,7 | 57,9 | 105,8 | 4,2 |
| Lysine | 5,66 | 5,72 | 101,1 | 7,5 | 31,9 | 31,1 | 97,6 | 5,6 | 54 | 52,9 | 98,0 | 4,8 |
| Methionine | 1,83 | 1,80 | 98,4 | 5,8 | 8,71 | 8,91 | 102,3 | 3,5 | 14,3 | 14,83 | 103,7 | 3,3 |
| Ornithine | 5,56 | 5,88 | 105,8 | 6,8 | 23,4 | 23,2 | 99,2 | 3,3 | 36,8 | 37,5 | 101,8 | 3,4 |
| Phenylalanine | 13 | 13,17 | 101,3 | 8,6 | 40,4 | 42,6 | 105,5 | 3,6 | 57,4 | 62,1 | 108,2 | 4,7 |
| Phosphoethanolamine | 1,08 | 1,12 | 103,7 | 7,5 | 6,64 | 6,92 | 104,2 | 4,2 | 11,8 | 12,43 | 105,3 | 5,0 |
| Phosphoserine | 0,865 | 0,974 | 112,5 | 4,5 | 4,32 | 4,34 | 100,3 | 3,8 | 7,19 | 7,34 | 102,1 | 2,7 |
| Pipecolic acid | 0,719 | 0,712 | 99,1 | 5,9 | 2,19 | 2,21 | 100,9 | 3,6 | 3,1 | 3,19 | 102,8 | 4,0 |
| Proline | 14,3 | 14,10 | 98,6 | 6,5 | 42,6 | 42,5 | 99,8 | 3,7 | 60,5 | 61,4 | 101,4 | 3,4 |
| Saccharopine | 0,594 | 0,599 | 100,8 | 8,3 | 1,26 | 1,26 | 100,2 | 5,4 | 1,55 | 1,63 | 104,9 | 5,8 |
| Sarcosine | 0,727 | 0,733 | 100,9 | 6,6 | 2,1 | 2,1 | 102,3 | 3,2 | 3 | 3,1 | 103,0 | 3,5 |
| Serine | 17,6 | 17,37 | 98,7 | 6,4 | 45,2 | 45,0 | 99,6 | 4,2 | 60,8 | 61,3 | 100,8 | 3,3 |
| Taurine | 7,78 | 7,68 | 98,7 | 6,3 | 27,5 | 27,7 | 100,9 | 3,6 | 40,4 | 41,1 | 101,8 | 3,7 |
| Threonine | 8,36 | 7,96 | 95,2 | 5,7 | 29,6 | 28,9 | 97,6 | 3,9 | 45,2 | 43,8 | 96,9 | 3,6 |
| Tryptophan | 2,87 | 2,95 | 102,9 | 5,1 | 12,1 | 12,5 | 102,9 | 4,4 | 19,6 | 20,5 | 104,5 | 3,4 |
| Tyrosine | 5,37 | 5,67 | 105,5 | 6,6 | 18,4 | 19,3 | 104,6 | 4,0 | 28,3 | 28,9 | 102,3 | 3,7 |
| Valine | 12 | 12,0 | 99,7 | 6,5 | 37,4 | 37,5 | 100,4 | 3,8 | 55,1 | 55,8 | 101,4 | 3,6 |

Table S 4: Interday precision (relative standard deviation, RSD (%)) and accuracy (AC (%)) of amino acids determined by 10-fold diluted quality controls (QC) 1, 2 and 3. Concentration (Conc., µmol/L), mean concentration (mean).

| **Interday (n=10)** | **QC1** | | | | **QC2** | | | | **QC3** | | | |
| --- | --- | --- | --- | --- | --- | --- | --- | --- | --- | --- | --- | --- |
| Amino acid | µmol/L | mean | AC | RSD | µmol/L | mean | AC | RSD | µmol/L | mean | AC | RSD |
| 1-Methylhistidine | 0,433 | 0,434 | 100,2 | 4,9 | 1,13 | 1,01 | 89,4 | 9,1 | 1,54 | 1,38 | 89,9 | 5,7 |
| α-Aminobutyric acid | 0,924 | 0,917 | 99,2 | 3,6 | 4,96 | 4,46 | 89,9 | 3,8 | 8,4 | 7,50 | 89,3 | 4,8 |
| β-Aminoisobutyric acid | 0,775 | 0,747 | 96,4 | 5,7 | 2,63 | 2,32 | 88,1 | 2,9 | 3,96 | 3,44 | 86,8 | 4,6 |
| 3-Methylhistidine | 1,17 | 1,19 | 101,5 | 6,6 | 5,24 | 4,81 | 91,9 | 6,2 | 8,51 | 7,80 | 91,6 | 7,1 |
| 4-Hydroxyproline | 1,47 | 1,44 | 97,8 | 2,5 | 9,07 | 7,97 | 87,9 | 1,8 | 15,8 | 13,81 | 87,4 | 3,6 |
| Acetyltyrosine | 1,05 | 1,00 | 95,1 | 3,6 | 4,66 | 4,04 | 86,7 | 4,6 | 7,35 | 6,37 | 86,6 | 4,6 |
| Adenosylhomocysteine | 0,723 | 0,681 | 94,2 | 6,2 | 2,57 | 2,22 | 86,6 | 4,6 | 3,8 | 3,30 | 86,7 | 3,8 |
| Alanine | 27,3 | 28,2 | 103,1 | 2,2 | 77,5 | 66,1 | 85,3 | 2,2 | 110,9 | 92,2 | 83,1 | 3,3 |
| Allo-Isoleucine | 1,3 | 1,3 | 99,8 | 1,9 | 8,61 | 7,76 | 90,1 | 2,9 | 14,8 | 13,26 | 89,6 | 4,1 |
| α-Aminoadipic acid | 0,532 | 0,521 | 97,9 | 2,2 | 1,3 | 1,2 | 88,7 | 2,8 | 1,82 | 1,6 | 88,5 | 4,1 |
| Anserine | 0,45 | 0,44 | 98,7 | 6,5 | 1,09 | 0,99 | 91,0 | 4,6 | 1,54 | 1,38 | 89,7 | 6,5 |
| Arginine | 4,91 | 4,89 | 99,6 | 3,9 | 18,9 | 16,8 | 88,7 | 4,0 | 28,3 | 25,2 | 89,0 | 4,1 |
| Argininosuccinic acid | 1,17 | 1,10 | 93,8 | 7,2 | 7,75 | 6,97 | 90,0 | 3,9 | 13 | 11,95 | 91,9 | 5,1 |
| Asparagine | 5,75 | 5,60 | 97,4 | 3,2 | 16 | 14,3 | 89,2 | 3,0 | 22,5 | 20,0 | 89,1 | 4,4 |
| Aspartic acid | 3,94 | 3,87 | 98,1 | 6,1 | 14,5 | 13,2 | 91,2 | 4,8 | 22,6 | 20,3 | 89,6 | 6,6 |
| β-Alanine | 1,98 | 1,87 | 94,6 | 3,3 | 6,25 | 5,51 | 88,1 | 3,5 | 9,29 | 8,11 | 87,3 | 4,1 |
| Carnosine | 0,858 | 0,861 | 100,3 | 6,5 | 2,56 | 2,27 | 88,6 | 5,9 | 3,87 | 3,48 | 90,0 | 6,8 |
| Citrulline | 2,28 | 2,24 | 98,2 | 3,1 | 10,8 | 9,4 | 86,9 | 1,8 | 18 | 15,3 | 85,2 | 4,2 |
| Cystathionine | 0,855 | 0,810 | 94,7 | 5,7 | 2,89 | 2,58 | 89,1 | 3,6 | 4,38 | 3,84 | 87,7 | 4,6 |
| Cysteine sulphate | 2,04 | 1,95 | 95,7 | 2,6 | 4,27 | 3,75 | 87,8 | 2,0 | 5,32 | 4,67 | 87,8 | 3,4 |
| Cystine | 1,14 | 1,06 | 93,0 | 6,7 | 7,7 | 6,8 | 88,7 | 3,0 | 13,5 | 11,9 | 88,1 | 4,9 |
| Ethanolamine | 1,65 | 1,63 | 98,8 | 8,1 | 11,5 | 10,2 | 89,0 | 7,1 | 20,3 | 18,1 | 89,1 | 7,1 |
| γ-Aminobutyric acid | 0,399 | 0,388 | 97,2 | 9,9 | 0,999 | 0,896 | 89,7 | 7,0 | 1,44 | 1,263 | 87,7 | 6,6 |
| Glutamine | 52,6 | 50,6 | 96,2 | 3,3 | 123,2 | 106,8 | 86,7 | 2,9 | 157,4 | 135,9 | 86,4 | 3,6 |
| Glutamic acid | 9,65 | 9,59 | 99,4 | 3,9 | 41,9 | 37,8 | 90,1 | 2,7 | 67,9 | 61,1 | 89,9 | 3,1 |
| Glycine | 33,2 | 32,9 | 99,0 | 4,2 | 84,3 | 72,5 | 86,0 | 2,4 | 114,7 | 99,7 | 86,9 | 4,0 |
| Histidine | 6,57 | 6,40 | 97,4 | 5,4 | 18,8 | 17,0 | 90,2 | 4,1 | 26,7 | 24,5 | 91,9 | 3,6 |
| Homocitrulline | 1,5 | 1,4 | 94,3 | 3,5 | 3,7 | 3,2 | 87,7 | 3,1 | 5,01 | 4,3 | 86,2 | 3,6 |
| Homocystine | 0,604 | 0,614 | 101,7 | 5,0 | 1,71 | 1,57 | 91,5 | 3,8 | 2,48 | 2,21 | 89,0 | 5,5 |
| Hydroxylysine | 0,721 | 0,702 | 97,4 | 5,8 | 2,13 | 1,87 | 88,0 | 5,6 | 3,08 | 2,71 | 87,9 | 5,0 |
| Isoleucine | 4,53 | 4,34 | 95,8 | 2,3 | 19,6 | 17,5 | 89,5 | 2,1 | 31,7 | 28,3 | 89,4 | 4,1 |
| Leucine | 11,2 | 11,7 | 104,1 | 4,3 | 37 | 34,2 | 92,5 | 3,3 | 54,7 | 50,1 | 91,6 | 2,8 |
| Lysine | 5,66 | 5,31 | 93,8 | 8,3 | 31,9 | 28,8 | 90,1 | 5,6 | 54 | 47,1 | 87,3 | 6,9 |
| Methionine | 1,83 | 1,80 | 98,1 | 2,4 | 8,71 | 7,91 | 90,8 | 2,0 | 14,3 | 13,03 | 91,1 | 3,5 |
| Ornithine | 5,56 | 5,25 | 94,4 | 8,2 | 23,4 | 21,1 | 90,4 | 4,2 | 36,8 | 33,3 | 90,5 | 3,8 |
| Phenylalanine | 13 | 13,14 | 101,1 | 3,4 | 40,4 | 37,2 | 92,2 | 3,5 | 57,4 | 52,9 | 92,1 | 2,8 |
| Phosphoethanolamine | 1,08 | 1,04 | 96,4 | 14,0 | 6,64 | 5,99 | 90,2 | 5,0 | 11,8 | 10,58 | 89,6 | 4,5 |
| Phosphoserine | 0,865 | 0,792 | 91,5 | 11,9 | 4,32 | 3,79 | 87,8 | 5,2 | 7,19 | 6,44 | 89,6 | 4,7 |
| Pipecolic acid | 0,719 | 0,721 | 100,3 | 2,7 | 2,19 | 2,00 | 91,4 | 2,4 | 3,1 | 2,80 | 90,4 | 3,2 |
| Proline | 14,3 | 14,4 | 100,4 | 2,2 | 42,6 | 38,8 | 91,2 | 3,1 | 60,5 | 54,2 | 89,6 | 4,3 |
| Saccharopine | 0,594 | 0,594 | 100,0 | 4,0 | 1,26 | 1,16 | 91,8 | 4,6 | 1,55 | 1,45 | 93,4 | 5,0 |
| Sarcosine | 0,727 | 0,717 | 98,6 | 2,6 | 2,1 | 1,91 | 91,0 | 2,4 | 3 | 2,69 | 89,8 | 3,4 |
| Serine | 17,6 | 17,2 | 97,5 | 6,0 | 45,2 | 40,2 | 89,0 | 4,7 | 60,8 | 53,8 | 88,5 | 4,6 |
| Taurine | 7,78 | 7,74 | 99,5 | 2,7 | 27,5 | 24,9 | 90,4 | 2,2 | 40,4 | 36,5 | 90,2 | 3,5 |
| Threonine | 8,36 | 8,13 | 97,3 | 7,3 | 29,6 | 26,7 | 90,1 | 4,7 | 45,2 | 40,2 | 88,9 | 5,1 |
| Tryptophan | 2,87 | 2,92 | 101,8 | 2,5 | 12,1 | 11,2 | 92,4 | 2,3 | 19,6 | 17,9 | 91,5 | 4,2 |
| Tyrosine | 5,37 | 5,55 | 103,4 | 2,4 | 18,4 | 17,0 | 92,3 | 2,2 | 28,3 | 25,3 | 89,4 | 3,3 |
| Valine | 12 | 11,99 | 99,9 | 2,3 | 37,4 | 33,8 | 90,3 | 2,8 | 55,1 | 49,3 | 89,5 | 3,7 |

Table S 5: Intraday precision (relative standard deviation, RSD (%)) and accuracy (AC (%)) of amino acids determined by 10-fold diluted calibration standards (Cal) 1, 2 and 3. Concentration (Conc., µmol/L), mean concentration (mean).

| **Intraday (n=10)** | **Cal1** | | | | **Cal2** | | | | **Cal3** | | | |
| --- | --- | --- | --- | --- | --- | --- | --- | --- | --- | --- | --- | --- |
|  | µmol/L | mean | AC | RSD | µmol/L | mean | AC | RSD | µmol/L | mean | AC | RSD |
| 1-Methylhistidine | 0,139 | 0,136 | 97,9 | 7,7 | 0,739 | 0,76 | 103,3 | 5,9 | 1,97 | 1,95 | 98,9 | 6,4 |
| α-Aminobutyric acid | 0,278 | 0,292 | 105,0 | 1,4 | 1,44 | 1,36 | 94,5 | 1,3 | 11,6 | 11,67 | 100,6 | 1,0 |
| β-Aminoisobutyric acid | 0,239 | 0,252 | 105,5 | 4,7 | 1,27 | 1,18 | 93,0 | 1,2 | 5,03 | 5,11 | 101,5 | 1,6 |
| 3-Methylhistidine | 0,395 | 0,40 | 101,6 | 6,4 | 2,05 | 2,01 | 98,2 | 6,6 | 12,3 | 12,33 | 100,3 | 6,7 |
| 4-Hydroxyproline | 0,48 | 0,50 | 105,1 | 2,1 | 2,4 | 2,26 | 94,4 | 1,5 | 21,7 | 21,81 | 100,5 | 1,5 |
| Acetyltyrosine | 0,234 | 0,25 | 106,5 | 3,8 | 1,81 | 1,67 | 92,3 | 2,8 | 9,77 | 9,89 | 101,3 | 2,9 |
| Adenosylhomocysteine | 0,152 | 0,159 | 104,6 | 4,8 | 1,25 | 1,17 | 94,0 | 5,0 | 4,97 | 5,04 | 101,4 | 4,7 |
| Alanine | 10,6 | 11,18 | 105,4 | 5,9 | 43 | 39,9 | 92,7 | 2,1 | 138,1 | 140,7 | 101,8 | 0,9 |
| Allo-Isoleucine | 0,178 | 0,19 | 105,0 | 1,5 | 2,38 | 2,25 | 94,4 | 1,5 | 20,8 | 20,92 | 100,6 | 0,9 |
| α-Aminoadipic acid | 0,29 | 0,302 | 104,2 | 1,7 | 0,782 | 0,74 | 94,5 | 2,1 | 2,35 | 2,38 | 101,3 | 1,1 |
| Anserine | 0,22 | 0,23 | 105,9 | 5,8 | 0,674 | 0,62 | 92,0 | 6,1 | 1,92 | 1,96 | 102,1 | 5,3 |
| Arginine | 0,666 | 0,70 | 104,9 | 5,6 | 9,41 | 8,8 | 94,0 | 3,0 | 36,9 | 37,4 | 101,4 | 4,1 |
| Argininosuccinic acid | 0,264 | 0,28 | 104,7 | 3,8 | 1,97 | 1,87 | 94,8 | 3,5 | 18,7 | 18,79 | 100,5 | 4,0 |
| Asparagine | 1,32 | 1,38 | 104,8 | 3,4 | 9,54 | 8,9 | 93,2 | 1,7 | 28,5 | 29,1 | 102,1 | 1,2 |
| Aspartic acid | 1,31 | 1,39 | 106,3 | 10,2 | 6,28 | 6,1 | 96,9 | 7,5 | 29,3 | 29,5 | 100,5 | 2,3 |
| β-Alanine | 0,54 | 0,56 | 104,4 | 5,3 | 3,11 | 2,93 | 94,3 | 1,8 | 12,1 | 12,25 | 101,3 | 1,7 |
| Carnosine | 0,491 | 0,518 | 105,5 | 5,2 | 1,18 | 1,10 | 93,5 | 4,2 | 5,04 | 5,09 | 101,0 | 3,2 |
| Citrulline | 0,776 | 0,80 | 103,7 | 4,2 | 3,61 | 3,5 | 95,8 | 3,8 | 24,1 | 24,2 | 100,5 | 2,6 |
| Cystathionine | 0,289 | 0,303 | 105,0 | 4,3 | 1,43 | 1,34 | 93,7 | 3,1 | 5,71 | 5,79 | 101,3 | 1,9 |
| Cysteine sulphate | 0,78 | 0,80 | 102,5 | 2,2 | 3,1 | 2,96 | 95,5 | 1,6 | 5,96 | 6,08 | 102,0 | 1,2 |
| Cystine | 0,353 | 0,38 | 107,9 | 5,1 | 1,85 | 1,72 | 93,1 | 3,6 | 18,6 | 18,71 | 100,6 | 2,0 |
| Ethanolamine | 0,761 | 0,82 | 107,1 | 14,0 | 2,9 | 2,9 | 98,6 | 11,7 | 28,3 | 28,3 | 100,1 | 1,2 |
| γ-Aminobutyric acid | 0,262 | 0,272 | 103,8 | 4,0 | 0,741 | 0,702 | 94,7 | 1,9 | 1,93 | 1,960 | 101,5 | 1,1 |
| Glutamine | 17,2 | 17,89 | 104,0 | 1,8 | 86,2 | 80,4 | 93,3 | 1,9 | 190,2 | 195,3 | 102,7 | 1,8 |
| Glutamic acid | 3,14 | 3,31 | 105,4 | 5,2 | 16,7 | 15,6 | 93,6 | 1,6 | 93,8 | 94,7 | 101,0 | 1,3 |
| Glycine | 14,1 | 15,05 | 106,8 | 4,0 | 51,2 | 48,4 | 94,6 | 2,1 | 142,5 | 144,3 | 101,3 | 1,2 |
| Histidine | 2,24 | 2,29 | 102,4 | 8,9 | 10,6 | 10,3 | 96,8 | 5,4 | 35,2 | 35,5 | 100,8 | 6,6 |
| Homocitrulline | 0,483 | 0,50 | 103,9 | 2,6 | 2,44 | 2,29 | 93,7 | 2,6 | 5,76 | 5,89 | 102,3 | 2,8 |
| Homocystine | 0,175 | 0,183 | 104,5 | 6,9 | 0,954 | 0,89 | 93,6 | 3,6 | 2,87 | 2,92 | 101,9 | 4,6 |
| Hydroxylysine | 0,229 | 0,255 | 111,4 | 15,2 | 1,2 | 1,151 | 95,9 | 4,4 | 3,92 | 3,962 | 101,1 | 2,3 |
| Isoleucine | 1,72 | 1,76 | 102,2 | 3,3 | 7,02 | 6,8 | 97,5 | 2,5 | 43,9 | 44,0 | 100,3 | 1,2 |
| Leucine | 3,92 | 3,99 | 101,7 | 2,6 | 19,4 | 19,0 | 97,8 | 2,0 | 74,5 | 74,9 | 100,5 | 1,6 |
| Lysine | 1,85 | 1,98 | 107,1 | 9,6 | 9,92 | 9,1 | 92,0 | 5,0 | 74,6 | 75,3 | 100,9 | 5,4 |
| Methionine | 0,567 | 0,60 | 105,4 | 1,8 | 3,02 | 2,83 | 93,8 | 1,5 | 20,3 | 20,46 | 100,8 | 1,2 |
| Ornithine | 1,12 | 1,21 | 108,2 | 13,1 | 10,2 | 9,6 | 94,0 | 4,2 | 49,8 | 50,4 | 101,1 | 1,9 |
| Phenylalanine | 2,49 | 2,57 | 103,3 | 8,0 | 23,8 | 22,7 | 95,3 | 3,5 | 76,9 | 77,9 | 101,3 | 2,1 |
| Phosphoethanolamine | 0,47 | 0,50 | 107,0 | 13,6 | 1,66 | 1,54 | 92,5 | 9,8 | 16,7 | 16,79 | 100,5 | 6,3 |
| Phosphoserine | 0,28 | 0,294 | 105,1 | 3,4 | 1,49 | 1,40 | 94,2 | 2,8 | 10,1 | 10,17 | 100,7 | 2,8 |
| Pipecolic acid | 0,176 | 0,182 | 103,5 | 2,4 | 1,26 | 1,20 | 95,2 | 2,0 | 4,06 | 4,11 | 101,3 | 1,5 |
| Proline | 3,97 | 4,14 | 104,2 | 2,0 | 24,5 | 23,1 | 94,2 | 1,3 | 77 | 78,3 | 101,6 | 1,4 |
| Saccharopine | 0,234 | 0,243 | 103,9 | 3,6 | 0,959 | 0,89 | 93,1 | 4,2 | 1,86 | 1,92 | 103,1 | 3,4 |
| Sarcosine | 0,238 | 0,246 | 103,3 | 4,2 | 1,2 | 1,1 | 95,5 | 2,0 | 3,92 | 4,0 | 101,2 | 1,5 |
| Serine | 5,94 | 6,13 | 103,2 | 3,0 | 28,6 | 27,3 | 95,3 | 1,9 | 75,5 | 76,7 | 101,5 | 0,9 |
| Taurine | 0,991 | 1,02 | 103,1 | 4,2 | 14,3 | 13,7 | 95,8 | 1,2 | 53,4 | 54,0 | 101,1 | 0,8 |
| Threonine | 2,96 | 3,22 | 108,8 | 4,9 | 13,6 | 12,1 | 89,1 | 6,2 | 58,6 | 59,8 | 102,1 | 2,8 |
| Tryptophan | 0,969 | 1,02 | 104,9 | 4,0 | 4,85 | 4,6 | 94,3 | 2,6 | 27,4 | 27,6 | 100,8 | 1,5 |
| Tyrosine | 1,9 | 1,98 | 104,2 | 2,6 | 9,44 | 8,9 | 94,6 | 1,2 | 37,5 | 37,9 | 101,1 | 0,8 |
| Valine | 4,78 | 5,0 | 104,3 | 1,4 | 19,3 | 18,3 | 94,6 | 1,4 | 71,9 | 72,7 | 101,2 | 1,1 |

Table S 6: Interday precision (relative standard deviation, RSD (%)) and accuracy (AC (%)) of amino acids determined by 10-fold diluted calibration standards (Cal) 1, 2 and 3. Concentration (Conc., µmol/L), mean concentration (mean).

| **Interday (n=10)** | **Cal1** | | | | **Cal2** | | | | **Cal3** | | | |
| --- | --- | --- | --- | --- | --- | --- | --- | --- | --- | --- | --- | --- |
|  | µmol/L | mean | AC | RSD | µmol/L | mean | AC | RSD | µmol/L | mean | AC | RSD |
| 1-Methylhistidine | 0,139 | 0,139 | 100,0 | 2,3 | 0,739 | 0,74 | 99,6 | 4,8 | 1,97 | 1,97 | 100,0 | 1,5 |
| α-Aminobutyric acid | 0,278 | 0,279 | 100,4 | 3,6 | 1,44 | 1,44 | 99,7 | 3,4 | 11,6 | 11,60 | 100,0 | 0,3 |
| β-Aminoisobutyric acid | 0,239 | 0,242 | 101,3 | 2,6 | 1,27 | 1,25 | 98,3 | 3,5 | 5,03 | 5,05 | 100,4 | 0,7 |
| 3-Methylhistidine | 0,395 | 0,395 | 100,0 | 5,0 | 2,05 | 2,05 | 100,0 | 5,7 | 12,3 | 12,30 | 100,0 | 0,8 |
| 4-Hydroxyproline | 0,48 | 0,48 | 99,6 | 4,0 | 2,4 | 2,41 | 100,5 | 4,3 | 21,7 | 21,69 | 100,0 | 0,4 |
| Acetyltyrosine | 0,234 | 0,236 | 100,9 | 3,0 | 1,81 | 1,78 | 98,5 | 3,2 | 9,77 | 9,79 | 100,2 | 0,5 |
| Adenosylhomocysteine | 0,152 | 0,155 | 102,0 | 3,4 | 1,25 | 1,23 | 98,5 | 4,1 | 4,97 | 4,99 | 100,4 | 0,9 |
| Alanine | 10,6 | 10,8 | 101,6 | 2,2 | 43 | 42,1 | 97,9 | 3,1 | 138,1 | 138,8 | 100,5 | 0,8 |
| Allo-Isoleucine | 0,178 | 0,177 | 99,4 | 3,8 | 2,38 | 2,40 | 100,6 | 4,0 | 20,8 | 20,79 | 99,9 | 0,4 |
| α-Aminoadipic acid | 0,29 | 0,29 | 100,7 | 2,2 | 0,782 | 0,78 | 99,4 | 3,5 | 2,35 | 2,36 | 100,2 | 0,8 |
| Anserine | 0,22 | 0,23 | 102,3 | 4,3 | 0,674 | 0,66 | 97,3 | 4,8 | 1,92 | 1,93 | 100,7 | 1,3 |
| Arginine | 0,666 | 0,671 | 100,8 | 2,8 | 9,41 | 9,3 | 99,0 | 3,6 | 36,9 | 37,0 | 100,2 | 0,9 |
| Argininosuccinic acid | 0,264 | 0,259 | 98,1 | 5,6 | 1,97 | 2,00 | 101,6 | 6,3 | 18,7 | 18,67 | 99,9 | 0,6 |
| Asparagine | 1,32 | 1,33 | 101,0 | 2,5 | 9,54 | 9,4 | 98,5 | 3,7 | 28,5 | 28,6 | 100,5 | 1,1 |
| Aspartic acid | 1,31 | 1,30 | 99,5 | 4,9 | 6,28 | 6,3 | 100,5 | 5,8 | 29,3 | 29,3 | 99,9 | 1,0 |
| β-Alanine | 0,54 | 0,54 | 100,7 | 3,5 | 3,11 | 3,07 | 98,7 | 4,4 | 12,1 | 12,14 | 100,3 | 1,0 |
| Carnosine | 0,491 | 0,492 | 100,2 | 2,7 | 1,18 | 1,18 | 99,9 | 3,3 | 5,04 | 5,04 | 100,0 | 0,5 |
| Citrulline | 0,776 | 0,769 | 99,1 | 3,4 | 3,61 | 3,7 | 101,1 | 3,9 | 24,1 | 24,1 | 99,9 | 0,5 |
| Cystathionine | 0,289 | 0,291 | 100,7 | 2,0 | 1,43 | 1,42 | 99,2 | 2,7 | 5,71 | 5,72 | 100,2 | 0,5 |
| Cysteine sulphate | 0,78 | 0,78 | 99,9 | 2,0 | 3,1 | 3,11 | 100,4 | 3,2 | 5,96 | 5,95 | 99,8 | 1,4 |
| Cystine | 0,353 | 0,36 | 102,0 | 2,9 | 1,85 | 1,82 | 98,2 | 3,3 | 18,6 | 18,63 | 100,2 | 0,3 |
| Ethanolamine | 0,761 | 0,733 | 96,3 | 5,1 | 2,9 | 3,0 | 104,1 | 5,1 | 28,3 | 28,2 | 99,7 | 0,4 |
| γ-Aminobutyric acid | 0,262 | 0,263 | 100,4 | 2,6 | 0,741 | 0,737 | 99,5 | 3,3 | 1,93 | 1,933 | 100,2 | 0,9 |
| Glutamine | 17,2 | 17,127 | 99,6 | 2,6 | 86,2 | 86,8 | 100,7 | 4,3 | 190,2 | 189,7 | 99,7 | 1,7 |
| Glutamic acid | 3,14 | 3,15 | 100,3 | 2,6 | 16,7 | 16,6 | 99,7 | 3,0 | 93,8 | 93,8 | 100,1 | 0,4 |
| Glycine | 14,1 | 15,8 | 112,1 | 5,9 | 51,2 | 51,5 | 100,6 | 4,1 | 142,5 | 140,5 | 98,6 | 2,0 |
| Histidine | 2,24 | 2,23 | 99,5 | 3,8 | 10,6 | 10,7 | 100,7 | 5,1 | 35,2 | 35,1 | 99,8 | 1,3 |
| Homocitrulline | 0,483 | 0,491 | 101,7 | 3,1 | 2,44 | 2,38 | 97,5 | 5,0 | 5,76 | 5,82 | 101,0 | 1,8 |
| Homocystine | 0,175 | 0,174 | 99,4 | 4,0 | 0,954 | 0,95 | 99,8 | 4,7 | 2,87 | 2,87 | 100,1 | 1,4 |
| Hydroxylysine | 0,229 | 0,231 | 100,9 | 3,8 | 1,2 | 1,180 | 98,3 | 5,4 | 3,92 | 3,938 | 100,5 | 1,4 |
| Isoleucine | 1,72 | 1,71 | 99,1 | 4,7 | 7,02 | 7,1 | 101,0 | 5,2 | 43,9 | 43,8 | 99,9 | 0,7 |
| Leucine | 3,92 | 3,89 | 99,2 | 2,8 | 19,4 | 19,6 | 101,0 | 3,5 | 74,5 | 74,3 | 99,8 | 0,8 |
| Lysine | 1,85 | 1,96 | 105,9 | 4,7 | 9,92 | 9,3 | 93,3 | 6,1 | 74,6 | 75,2 | 100,7 | 0,6 |
| Methionine | 0,567 | 0,573 | 101,1 | 3,2 | 3,02 | 2,99 | 98,9 | 3,8 | 20,3 | 20,33 | 100,1 | 0,5 |
| Ornithine | 1,12 | 1,14 | 101,3 | 4,6 | 10,2 | 10,0 | 98,2 | 5,8 | 49,8 | 50,0 | 100,3 | 1,1 |
| Phenylalanine | 2,49 | 2,47 | 99,2 | 3,1 | 23,8 | 24,0 | 101,0 | 4,1 | 76,9 | 76,7 | 99,7 | 1,2 |
| Phosphoethanolamine | 0,47 | 0,50 | 106,8 | 4,7 | 1,66 | 1,53 | 92,4 | 5,5 | 16,7 | 16,79 | 100,6 | 0,4 |
| Phosphoserine | 0,28 | 0,30 | 105,4 | 3,7 | 1,49 | 1,40 | 94,1 | 4,3 | 10,1 | 10,18 | 100,7 | 0,5 |
| Pipecolic acid | 0,176 | 0,176 | 100,0 | 2,9 | 1,26 | 1,27 | 100,8 | 3,6 | 4,06 | 4,05 | 99,8 | 1,0 |
| Proline | 3,97 | 3,96 | 99,8 | 2,7 | 24,5 | 24,5 | 100,2 | 3,7 | 77 | 77,0 | 99,9 | 1,1 |
| Saccharopine | 0,234 | 0,238 | 101,7 | 2,7 | 0,959 | 0,94 | 97,8 | 4,4 | 1,86 | 1,88 | 101,0 | 1,9 |
| Sarcosine | 0,238 | 0,239 | 100,4 | 3,7 | 1,2 | 1,2 | 100,2 | 4,2 | 3,92 | 3,9 | 99,9 | 1,1 |
| Serine | 5,94 | 5,90 | 99,2 | 3,2 | 28,6 | 28,9 | 101,1 | 4,6 | 75,5 | 75,2 | 99,6 | 1,5 |
| Taurine | 0,991 | 0,981 | 99,0 | 2,9 | 14,3 | 14,5 | 101,3 | 3,7 | 53,4 | 53,2 | 99,7 | 1,0 |
| Threonine | 2,96 | 2,97 | 100,2 | 3,0 | 13,6 | 13,6 | 99,7 | 3,8 | 58,6 | 58,6 | 100,1 | 0,7 |
| Tryptophan | 0,969 | 0,970 | 100,1 | 3,3 | 4,85 | 4,8 | 99,9 | 3,8 | 27,4 | 27,4 | 100,0 | 0,6 |
| Tyrosine | 1,9 | 1,91 | 100,3 | 2,7 | 9,44 | 9,4 | 99,6 | 3,4 | 37,5 | 37,5 | 100,1 | 0,7 |
| Valine | 4,78 | 4,76 | 99,5 | 2,8 | 19,3 | 19,4 | 100,6 | 3,5 | 71,9 | 71,8 | 99,9 | 0,8 |
